# Podoplanin is indispensable for cell motility and platelet-induced epithelial-to-mesenchymal transition-related gene expression in esophagus squamous carcinoma TE11A cells

**DOI:** 10.1186/s12935-020-01328-2

**Published:** 2020-06-23

**Authors:** Nobuo Watanabe, Masako Kidokoro, Makiko Tanaka, Shigeaki Inoue, Tomoatsu Tsuji, Hisako Akatuska, Chisa Okada, Yumi Iida, Yoshinori Okada, Yusuke Suzuki, Takehito Sato, Takashi Yahata, Noriaki Hirayama, Yoshihide Nakagawa, Sadaki Inokuchi

**Affiliations:** 1 Department of Emergency and Critical Care Medicine, Tokai University School of Medicine, 143 Shimokasuya, Isehara, Kanagawa 259-1193 Japan; 2 Department of Host Defense Mechanism, Tokai University School of Medicine, 143 Shimokasuya, Isehara, Kanagawa 259-1193 Japan; 3 Support Center for Medical Research and Education, Tokai University, 143 Shimokasuya, Isehara, Kanagawa 259-1193 Japan; 4 Research Center for Regenerative Medicine, Tokai University School of Medicine, 143 Shimokasuya, Isehara, Kanagawa 259-1193 Japan; 5 Institute of Advanced Biosciences, Tokai University, 411 Kitakaname, Hiratsuka, Kanagawa 259-1292 Japan

**Keywords:** Metastasis, Platelet, Esophageal squamous cell carcinoma, Crispr-Cas-9, Knockdown

## Abstract

**Background:**

The transmembrane glycoprotein podoplanin (PDPN) is upregulated in some tumors and has gained attention as a malignant tumor biomarker. PDPN molecules have platelet aggregation-stimulating domains and, are therefore, suggested to play a role in tumor-induced platelet activation, which in turn triggers epithelial-to-mesenchymal transition (EMT) and enhances the invasive and metastatic activities of tumor cells. In addition, as forced PDPN expression itself can alter the propensity of certain tumor cells in favor of EMT and enhance their invasive ability, it is also considered to be involved in the cell signaling system. Nevertheless, underlying mechanisms of PDPN in tumor cell invasive ability as well as EMT induction, especially by platelets, are still not fully understood.

**Methods:**

Subclonal TE11A cells were isolated from the human esophageal squamous carcinoma cell line TE11 and the effects of anti-PDPN neutralizing antibody as well as PDPN gene knockout on platelet-induced EMT-related gene expression were measured. Also, the effects of PDPN deficiency on cellular invasive ability and motility were assessed.

**Results:**

PDPN-null cells were able to provoke platelet aggregation, suggesting that PDPN contribution to platelet activation in these cells is marginal. Nevertheless, expression of platelet-induced EMT-related genes, including vimentin, was impaired by PDPN-neutralizing antibody as well as PDPN deficiency, while their effects on TGF-β-induced gene expression were marginal. Unexpectedly, PDPN gene ablation, at least in either allele, engendered spontaneous N-cadherin upregulation and claudin-1 downregulation. Despite these seemingly EMT-like alterations, PDPN deficiency impaired cellular motility and invasive ability even after TGF-β-induced EMT induction.

**Conclusions:**

These results suggested that, while PDPN seems to function in favor of maintaining the epithelial state of this cell line, it is indispensable for platelet-mediated induction of particular mesenchymal marker genes as well as the potentiation of motility and invasion capacity.

## Background

Podoplanin (PDPN, also known as aggrus) is a type I glycoprotein expressed selectively in certain tissues, such as lymphatic endothelial cells and podocytes in the kidney [1–3]. It plays a critical role in embryonic development, as well as the postnatal maintenance of certain organs, including the lymphatic vascular system [2, 4]. Previously, we [5] and others [6, 7] have demonstrated that elevated levels of PDPN in the tumor tissues of patients with esophageal squamous carcinoma, especially in cells at the invasive edge, are correlated with poor prognosis after esophagectomy. This correlation has also been observed in other squamous cell carcinoma, as well as glioma, mesothelioma, and melanoma, establishing PDPN as a malignant tumor biomarker [8, 9].

The epithelial-to-mesenchymal transition (EMT) is an important process in tumor invasion and metastasis. EMT results in the loss of the epithelial cell machinery responsible for the formation of tight junctions and adhesion junctions, including E-cadherin and claudin-1, with a concomitant induction of mesenchymal markers, such as N-cadherin and vimentin [10, 11]. As a result, tumor cells lose cell-to-cell contacts and polarity and gain cell motility, such that they can transverse vascular walls and colonize distant organs. Previous in vitro studies demonstrated that the enforced expression of PDPN itself induced EMT, including the expression of N-cadherin, and enhanced cellular motility [12, 13]. In contrast, the siRNA-mediated downregulation of PDPN in high-PDPN-expressing cancer cells conversely resulted in a mesenchymal-to-epithelial transition in some marker genes, decreasing cell motility and invasive capacity [6, 14, 15]. However, other studies have demonstrated that forced PDPN expression stimulates tumor cell migration/invasion as clusters without inducing EMT (collective cell migration) [16]. In any case, the levels of PDPN seem to contribute positively to the malignant characteristics of tumor cells.

Tumor-induced platelet activation has been suggested to play a pivotal role in tumor metastasis and invasion [17, 18]. Experimental evidence demonstrated that tumor-activated platelets induce EMT and support tumor cell proliferation [15, 17, 19, 20]. In addition, the attachment of platelets onto the surface of tumor cells is thought to protect the tumor cells from attack by natural killer cells [21] and subvert T cell immunity against the tumor cells [22]. Among various possible tumor cell-platelet interactions, the interaction of PDPN with C-type-lectin-like-2 (CLEC-2) on the platelet surface has gained significant attention. PDPN molecules have 4 tandem repeats of platelet aggregation-stimulating domains (PLAG 1–4) [23, 24], of which the latter two interact with CLEC-2 [23, 25, 26]. The PDPN-CLEC-2 interaction triggers platelet activation, resulting in platelet aggregation and the release of a range of granule-stored molecules, including growth factors, such as epidermal growth factor (EGF) and transforming growth factor-β (TGF-β), as well as immune-modulating small molecules, such as sphingosine 1-phosphate (S1P) [4, 15, 19]. In highlighting the crucial role of PDPN-CLEC-2 interaction, the administration of a PDPN-neutralizing antibody or a small-molecule inhibitor of the PDPN-CLEC-2 interaction inhibits lung metastasis in mouse models [27–29]. Nevertheless, beyond the potential interaction with CLEC-2, the roles of PDPN in platelet-induced tumor cell EMT and invasibility remains poorly understood.

To gain insights into the malignant nature of PDPN in esophageal squamous cell carcinoma, in this study, we investigated the role of PDPN in EMT progression and invasion ability, especially focusing on the interaction with platelets, in human esophageal squamous cell carcinoma TE11 cells. To this end, using a subclone of TE11 cells (TE11A cells), we generated PDPN-knockout clonal cells and addressed the mechanistic aspect of EMT progression and the alteration of the invasion ability after treatment with human platelets. Herein, we describe the role of PDPN in platelet-induced EMT progression and invasion in these cells, as well as the unexpected characteristics of PDPN deficiency.

## Materials and methods

### Reagents

Recombinant human TGF-β was purchased from Peprotech (Rocky Hill, NJ, USA). β-galactosidase-conjugated streptavidin (SA-β-gal) was purchased from Life Technologies (Carlsbad, CA, USA) and was reconstituted with distilled water to 2 mg/mL, according to the manufacturer’s instructions. 4-methylumbelliferyl β-d-galactopyranoside (4MUG) and anti-β-actin antibody were purchased from Sigma Aldrich (St Louis, MO, USA). Crystal violet and Sepasol were purchased from Nacalai Tesque (Kyoto, Japan). Anti-PDPN antibodies (clone NZ-1) were purchased from AngioBio (Del Mar, CA, USA) for western blotting and from Medical & Biological Laboratories (Nagoya, Japan) for bioassays. The monoclonal anti-vimentin antibody (V9) was purchased from Dako (Glostrup, Denmark), rabbit polyclonal anti-vimentin antibody (10366-1-AP) was from Proteintech (Rosemont, IL, USA), anti-E-cadherin antibody (4A2C7) was purchased from Thermo Fisher Scientific (Waltham, MA, USA), and anti-N-cadherin antibody (IAR06) was purchased from Leica Biosystems (Nussloch, Germany). SB431542 and *E. coli* DH5α were obtained from Wako Chemicals (Tokyo, Japan). pSpCas9(BB)-2A-Puro was obtained from Addgene (Watertown, MA, USA).

### Cells and subcloning

TE11 cells were obtained from the RIKEN Cell Bank (Tsukuba, Japan) and were cultured in a low-glucose DMEM medium (Nacalai 08456-65) containing 10% (v/v) fetal calf serum (Gibco 10091) and antibiotics. TE11 cells were subcloned by limiting dilution in general tissue culture-treated 96 well plates (Falcon 353072). A single clone was obtained (TE11A cells), which was subsequently used throughout this study.

### CRISPR-Cas-9-mediated generation of PDPN knockout cells

The CRISPR-Cas-9 method was used to silence the PDPN gene. The all-in-one type targeting vector, pSpCas9(BB)-2A-Puro [30], was used with the following guide RNA sequences [19]: forward, 5′-CACCGGAAGATGACACTGAGACTAC-3′ and reverse, 5′-AAACGTAGTCTCAGTGTCATCTTCC-3′. The annealed oligo DNA and the targeting vector plasmid were digested with Bbs 1 and the two fragments were ligated. The plasmid was amplified in *E. coli* DH5α and purified using a FastGene Midi kit (NIPPON Genetics, Tokyo, Japan), according to the manufacturer’s instructions. The DNA sequences covering the insertion region were confirmed by sequencing.

TE11 subclone cells (parental TE11A cells), as established above, were transfected with the PDPN-targeted CRISPR-Cas9 plasmid vector by electroporation using a Neon Transfection System (Thermo Fisher Scientific). After 2 days of recovery culture, plasmid-transfected cells were selected with puromycin (0.5 µg/mL). After confirming the presence of knockdown cells in the bulk cell population by T7E1 assay, cells were cloned by limiting dilution in 96-well plates. Two clones, clone 2 and clone 4, were selected for further experiments.

Genomic DNA was isolated from the clones using a DNeasy Blood and Tissue kit (Qiagen, Hilden, Germany), according to the manufacturer’s instructions. PCR amplification of the PDPN gene encompassing the CRISPR target site was performed using the following primers: forward, 5′-AGATGTACGTCCCCTGTCCA-3′ and reverse, 5′-ACACCAGCTAAGTGGACGAAA-3′. After separation by agarose gel electrophoresis, the upper and lower bands were resected and the DNA was isolated using a NucleoSpin Gel and PCR Clean-up kit (Macherey–Nagel, Duren, Germany), according to the manufacturer’s instructions. DNA sequencing was performed using the abovementioned PCR primers.

### Immunofluorescence microscopy, FACS and ELISA analysis of cell surface PDPN expression

Cells cultured in Lab-Tek 4 chamber culture slides (Nalge Nunc) were fixed with 4% paraformaldehyde in PBS for 15 min at room temperature (RT), and blocked for 1 h with a blocking reagent (1% skim milk, 0.75% glycine in PBS). PDPN was stained with anti-PDPN antibody (NZ-1, 2.5 µg/mL), followed by DyLight594-labeled anti-rat IgG secondary antibody (500×, BioLegend) and Hoechst 33342 nuclear staining. Images were observed using an Axio Imager M2 (ZEISS). For cell ELISA, cells grown in 96-well plates were washed with PBS, fixed with 0.1% (v/v) glutaraldehyde (GA) in PBS for 5 min at RT, and blocked for 30 min with a blocking reagent (0.1% BSA, 0.2% gelatin, 0.75% glycine, 0.01% NaN_3_ in PBS), as described previously [31]. The cells were then incubated first with an anti-PDPN antibody (NZ-1, 0.25 µg/mL) in 0.1% BSA/PBS for 1 h, then with a biotinylated anti-rat IgG secondary antibody for 1 h, and finally with SA-β-gal in 0.1% BSA/PBS for 30 min. After washing the wells three times with 0.1% BSA/PBS, an enzyme reaction was performed with 100 µL of substrate solution (100 µM 4MUG, 1.5 mM MgCl_2_, 5 mM 2-mercaptoethanol in PBS) at 30 °C for 30 min. The reaction was terminated by the addition of 100 µL of 0.5 M Na_2_CO_3_ and the fluorescence intensity (excitation (Ex): 365 nm, emission (Em): 450 nm) was measured using a SpectraMax Plus plate reader (Molecular Devices, Sunnyvale, CA, USA). Wells devoid of cells, but otherwise treated with the same experimental procedure, were used as blanks and the mean fluorescence value of these wells was subtracted from the value of the sample wells. After fluorescence measurement, the plates were washed with PBS and GA-fixed cells in the wells, then stained with 100 µL of 0.5% (w/v) crystal violet and 1% ethanol for 30 min before finally washing three times with tap water. The stained cells were then dissolved in 150 µL of 1% (w/v) sodium deoxycholate solution, and their absorbance was measured at 550 nm in a plate reader (SpectraMax) [31].

For flowcytometry analysis, cells were harvested by trypsinization and suspended in 0.1% BSA in PBS. Cell suspension aliquots (5 × 10^5^ cells/50 µL) were first stained with an anti-PDPN antibody (NZ-1, 2 µg/mL) for 1 h, then with an APC-conjugated anti-rat IgG secondary antibody (200×, BioLegend) for 1 h, and analyzed by FACS (Fortessa; BD Biosciences).

### Preparation of platelets and cell treatment

The study using human blood was approved by the clinical research committee of Tokai University (19R067). Blood was collected from a healthy volunteer using evacuated blood collection tubes containing 3.2% (w/v) trisodium citrate. Tubes were centrifuged at 150×*g* for 10 min at RT and platelet-rich plasma (PRP) was obtained. PRP samples were diluted > threefold with wash buffer (0.2% [w/v] disodium citrate, 1% [w/v] BSA in PBS) and centrifuged at 500×*g* for 10 min at RT. The pellets were resuspended in the same volume of wash buffer, then sedimented again under the same centrifugation conditions. The platelet pellets were resuspended in a small volume of wash buffer and filtrated through a 35-µm cell strainer (BD-Falcon, 352235) to remove aggregates. Platelet density was adjusted at 2–4 × 10^8^/mL, and stored at RT until use.

TE11A parental cells or their clone cells were grown until confluence in 24-well plates. On the day of the experiment, the medium was replaced with 220 µL/well of fresh medium. Just before the initiation of co-culture with platelet, EGTA·4Na (typically 2 mM) was added to the wells, followed by 15 µL/well of the platelet suspensions. After incubation for 5 or 20 h, the cells were washed with PBS to remove platelets and lysed in Sepasol for PCR analysis. For western blotting, the experiments were scaled up proportionally to 12- or 6-well plates, and, at the end of the treatment, wells were washed three times with PBS to eliminate platelets. In some experiments, treatment with platelets or TGF-β was continued for 3 days, and twofold volume of fresh medium was additionally supplemented at 24 h after treatment.

### Platelet aggregation assay

Platelet aggregation kinetics were measured at 37 °C on a 4-chamber platelet aggregometer (PA-200; Kowa, Tokyo, Japan). PRP samples, obtained as described above by centrifugation at 150×*g*, were diluted threefold with PBS. An aliquot of 300 µL was added to the assay cuvettes. After recording the baseline for 30 s, 10 µL of a TE11A cell suspension (1–3 × 10^7^/mL) was added and the turbidity was measured for 20 min. In each run, PBS (vehicle), parental cells, clone 2 cells, and clone 4 cells were assayed simultaneously, with the same number of cells as the initiator. The increase in turbidity due to the addition of cell suspensions was subtracted from the measurements. TE11A cell clones were harvested by trypsinization under identical conditions.

### Real-time PCR and PCR

The mRNA was extracted from the cells by using Sepasol. Complementary DNA (cDNA) was synthesized using a High-Capacity cDNA Reverse Transcription kit (Life Technologies), according to the manufacturer’s instructions. Real-time PCR was performed using KOD SYBR qPCR Mix (Toyobo, Osaka, Japan) on an ABI 7500 real-time thermal cycler (Applied Biosystems, Foster City, CA, USA). The 2^−ΔΔCt^ method was used to calculate the fold change in the expression of each gene of interest. GADPH was used as an internal control. The sequences of the PCR primers used in this study are as follows: PDPN, forward: 5′-TGACTCCAGGAACCAGCGAAG-3′ and reverse: 5′-GCGAATGCCTGTTACACTGTTGA-3′; E-cadherin, forward: 5′-GAAGGTGACAGAGCCTCTGGAT-3′ and reverse: 5′-GATCGGTTACCGTGATCAAAATC-3′; N-cadherin, forward: 5′-CGGGTAATCCTCCCAAATCA-3′ and reverse: 5′-CTTTATCCCGGCGTTTCATC-3′; PAI-1, forward: 5′-GCACCACAGACGCGATCTT-3′ and reverse: 5′-ACCTCTGAAAAGTCCACTTGC-3′; claudin-1, forward: 5′-GCGATATTTCTTCTTGCAGGTC-3′ and reverse: 5′-TTCGTACCTGGCATTGACTGG-3′; vimentin, forward: 5′-TGTCCAAATCGATGTGGATGTTTC-3′ and reverse: 5′-TTGTACCATTCTTCTGCCTCCTG-3′; and GAPDH, forward: 5′-TGCACCACCAACTGCTTAGC-3′ and reverse: 5′-GGCATGGACTGTGGTCATGAG-3′. The cycling conditions were as follows: stage 1, 95 °C for 20 s; stage 2, 40 consecutive cycles of 95 °C for 3 s, then 60 °C for 15 s.

In some experiments, the full-length open-reading frame (ORF) of the mRNA of interest was amplified by RT-PCR using KOD-Fx Neo (Toyobo) and analyzed by agarose gel electrophoresis. The following primer sets were used for these experiments: GAPDH, forward: 5′-ATGGGGAAGGTGAAGGTCGGAGTCA-3′ and reverse: 5′-TTACTCCTTGGAGGCCATGTGGG-3′; and PDPN, forward: 5′-ATGTGGAAGGTGTCAGCTCTGCTCT-3′ and reverse: 5′-TTAGGGCGAGTACCTTCCCGACATT-3′. The PCR conditions were as follows: 95 °C for 2 min, followed by 20–30 cycles of amplification (95 °C for 10 s, 58 °C for 30 s, and 68 °C for 45 s) and 68 °C for 5 min.

### Western blot analysis

Cells were lysed on ice in a lysis buffer (0.5% [v/v] Triton X-100, 150 mM NaCl, 50 mM Tris–HCl [pH 7.4]) containing a protease inhibitor cocktail (Roche, Basel, Switzerland) and were then sonicated. The protein concentrations were determined using a DC Protein Assay kit (BioRad, Hercules, CA, USA). Equal amounts of protein were separated by SDS-PAGE using a 10% polyacrylamide gel and were transferred onto PVDF membranes. The membranes were probed with the appropriate primary antibody, followed by horseradish peroxidase (HRP)-conjugated secondary antibody. Chemiluminescence reactions were performed using an HRP substrate (Millipore, Billerica, MA, USA) and the signal was recorded with an image analyzer (ATTO, Tokyo, Japan).

### Wound-healing assay

TE11A cell clones were plated in 24-well plates at a density of 1 × 10^5^ cells/well. The next day, when the cells reached near-confluence, a scratch was made in each well using a 10 µL pipette tip. Cells were maintained in the presence or absence of TGF-β (10 ng/mL) for 2 days and swaths were monitored by microscopy (Axio Vert.A1; Carl ZEISS, Jena, Germany). Two positions on each swath were chosen and recorded before and after the 2-day culture. The areas of the swath in the microscope images were quantitated using Image J software (National Institutes of Health, Bethesda, MD, USA).

### Invasion assay

TE11 clone cells were treated with TGF-β (20 ng/mL) or platelets plus EGTA (2 mM) for 24 h, followed by supplementation with fresh medium and an additional culturing period of 2 days. Cells were harvested by trypsinization, then washed with PBS containing 1% BSA. Cells were then suspended in serum-free DMEM medium, and added to trans-well matrigel invasion assay chambers with 8-µm pore membranes (2 × 10^5^ cells/chamber; Corning 354480). The outer wells were filled with 10% serum-containing medium. After 48 h, the cells and matrigel inside the chamber were completely removed with twisted Kim Wipe papers, and invaded cells outside the membrane were fixed with 0.1% glutaldehyde in PBS for 10 min at RT. The cell chambers were reinstated in 24-well plates with 100 µL of 1 µg/ml DAPI in DABCO mounting medium placed between the bottom of the membrane and the well. Cells were counted by ArrayScan VTI (Thermo Fisher, Pittsburgh, PA, USA) with a 10× objective lens. For each well, 196 pictures were taken and the number of DAPI-positive cells was counted according to the Target Activation BioApplication program of the ArrayScan.

### Statistical analysis

Data were analyzed by one-way analysis of variance (ANOVA), followed by Tukey’s test, or unpaired Student’s t-test using Prism 5 software (GraphPad, San Diego, CA, USA). Two-way repeated ANOVA and Bonferroni’s test were performed to determine the main effects of the factors and clones, as well as their interaction. P < 0.05 was considered statistically significant.

## Results

### Subcloning of TE11 cell line

**Fig. 1:**
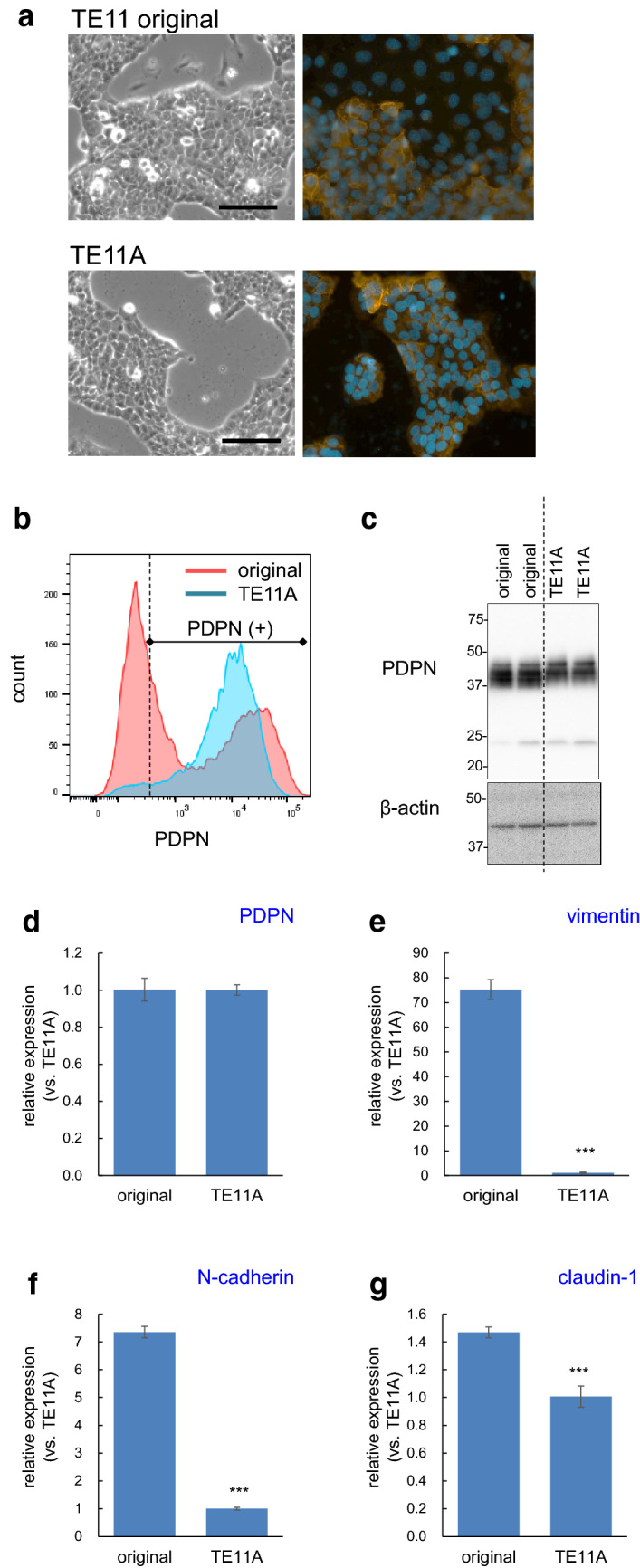
Comparison of sub-cloned TE11A and original TE11 cell population. **a** Proliferation phase morphology of the original TE11 and TE11A cells grown in culture dishes. In TE11 cells, some cells departed from the epithelial cell-like congregates. Scale bar: 100 µm. The right-side images show immunofluorescent staining of PDPN (orange) and nuclei (blue) at the same magnification. **b** FACS analysis of the cell-surface PDPN expression. The TE11 cell population is shown in red and subcloned TE11A cells in blue. The right side of dotted line represents the PDPN expressing cell population. **c** Western blot analysis of total PDPN. **d**–**g** Real-time PCR analysis of basal PDPN and EMT-related gene expression levels. The expression levels in TE11 cells were expressed relative to those in TE11A cells. The values are mean ± SEM of four culture wells in one representative experiment. ***P < 0.001 vs. original TE11 cells by unpaired Student’s t-test

It has been reported that TE11 cells are heterogeneous in terms of the levels of PDPN expression, even containing PDPN-negative populations [7]. Therefore, we subcloned TE11 cells by limiting dilution and obtained a PDPN-positive clonal cell (TE11A cells) for mechanistic studies. Microscopic observation showed that the original TE11 cells were consisting of congregated epithelial-like cells and some scattered large irregular-shaped cells, whereas TE11A cells comprised exclusively congregated epithelial-like cells (Fig. 1a). Immunofluorescence microscopy revealed that PDPN expression was confined to the congregated epithelial-like cells in either cell population. FACS analysis demonstrated that TE11A cells were exclusively PDPN-positive, while the original TE11 cells contained both low-expressing or negative and high-expressing cells (Fig. 1b), in agreement with the results of a previous report [7]. It is notable that peak level of PDPN expression was much hinger in the original TE11 cells than in TE11A cells. Nevertheless, due to mathematical averaging of the bimodal distribution, the mean fluorescence intensity for PDPN of TE11 cells was almost the same as that of TE11A cells (12,679 ± 574 vs. 13,046 ± 383, respectively), indicating that the cell-surface PDPN expression levels were almost the same between the two TE11 cells. Western blot analysis also demonstrated that the PDPN protein levels in both TE11 and TE11A cells were similar (Fig. 1c). We also checked the steady-state PDPN and EMT-related gene expression levels by real-time PCR. Consistent with FACS and western blot analysis, PDPN mRNA levels were also comparable between the two cell lines (Fig. 1d). Nevertheless, vimentin and N-cadherin levels were significantly different, with vimentin and N-cadherin levels in TE11 cells being 70- and sevenfold, respectively, higher than those in TE11A cells (Fig. 1e, f). However, the epithelial marker claudin-1 mRNA level was not hugely different (Fig. 1g). Overall, TE11A cells are relatively more skewed to epithelial-like phenotype than TE11 cells.

### Comparison of platelet- and TGF-β-induced EMT-related gene expression in TE11A cells

**Fig. 2:**
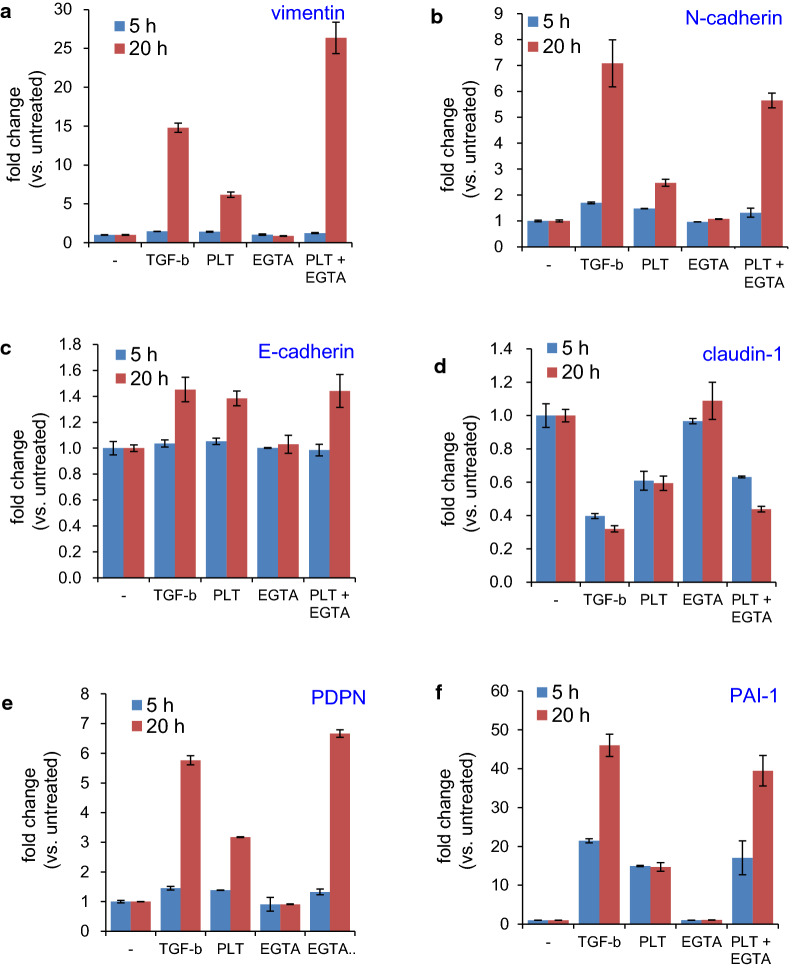
Time dependence of PLT- and TGF-β-induced expression of EMT-related genes in TE11A cells. TE11A cells at confluence in 24-well plates were treated with TGF-β (20 ng/mL) or platelets (~ 2 × 10^7^/mL), EGTA (2 mM), or platelets plus EGTA for 5 h or 20 h, and RNA was extracted. Expression of the EMT-related genes, vimentin (**a**), N-cadherin (**b**), E-cadherin (**c**), claudin-1 (**d**), PDPN (**e**), and *PAI*-*1* (**f**) was analyzed by real-time PCR. Values are mean ± range of duplicate culture wells from one representative experiment. Note the presence of EGTA (2 mM) potentiated gene alterations by platelets as compared with platelets alone, the presence of ETGA alone had no effect on basal expression levels of those genes

A previous study demonstrated that TGF-β, a major releasate from activated platelets, induces the expression of EMT-related genes in TE11 cells [14]. However, whether this cell line can respond to platelets themselves has not been investigated. Therefore, we assessed the treatment conditions with platelets in TE11A cells. To minimize the non-specific activation of platelets in culture medium before their interaction with the TE11A cell monolayers, the effect of Ca^2+^ chelation in the medium by EGTA was measured. Inclusion of EGTA at 2 mM, almost equimolar with the concentration of free Ca^2+^ in the medium, resulted in the potentiation of platelet-dependent expression of the mesenchymal marker gene, vimentin (Additional file 1: Fig. S1A). However, excess EGTA (> 3 mM) resulted in an almost complete inhibition of gene expression. This effect of EGTA was specific for stimulation by platelets, and EGTA had no effect on TGF-β-induced vimentin expression (Additional file 1: Fig. S1B). Therefore, 2 mM EGTA, which is expected to keep free Ca^2+^ concentration in the medium at sub-micromolar levels and itself has no effect on EMT-related gene expression (see EGTA alone in Fig. 2), was included in the media throughout this study when cells were treated with platelets.

Incubation of TE11A cells with platelets alone or with EGTA for 5 h had marginal effects on the mesenchymal marker genes, vimentin, and N-cadherin. However, after 20 h, the expression levels of both genes were elevated (Fig. 2a, b). In contrast, the downregulation of the epithelial marker gene, claudin-1, was observed as early as 5 h after treatment, and remained at this reduced level after 20 h (Fig. 2d). However, the expression of another epithelial marker, E-cadherin, was not reduced, but slightly increased by platelets after 20 h (Fig. 2c). Regardless of the direction of regulation, the kinetics of gene expression induced by platelets were essentially the same as those induced by TGF-β. Both TGF-β and the platelets also induced PDPN and pro-metastatic *PAI*-*1* [32] expression (Fig. 2e, f). It is worth noting that, unlike vimentin, N-cadherin, and PDPN, the induction of *PAI*-*1* was observed as early as 5 h after TGF-β or platelet treatment. Thus, platelets induce TE11A cells to express EMT-related genes in a similar fashion as TGF-β. In either case, however, EMT-related gene induction in TE11A cells is not associated with the “cadherin switch.”

To assess the contribution of TGF-β to the regulation of platelet-induced EMT gene expression, the effect of the ALK5 (TGF-β receptor kinase) inhibitor, SB431542, was measured. As reported previously [14], SB431542 completely suppressed TGF-β-induced changes in EMT gene expression in TE11A cells (Additional file 1: Fig. S3). Surprisingly, SB431542 also completely suppressed all EMT gene expression alterations induced by platelets. These results suggested that, at least at the mRNA expression level, the bioactive effects of platelets are largely mediated by TGF-β secreted from activated platelets, as suggested by experiments of other tumor cells treated with murine platelets [15, 17].

### Role of PDPN in platelet- or TGF-β-induced regulation of EMT genes in TE11A cells

**Fig. 3:**
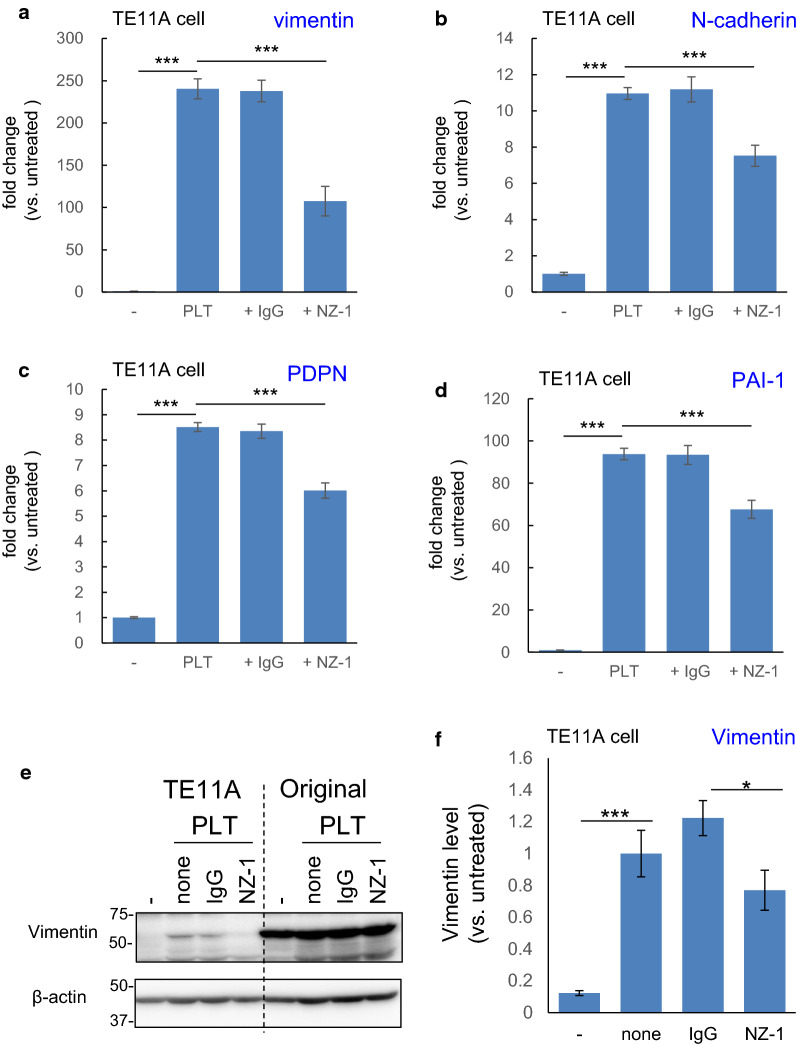
Effect of the PDPN-neutralizing antibody, NZ-1, on PLT-induced expression of EMT-related genes in TE11A cells. TE11A cells at confluence in 24-well plates were preincubated with NZ-1 or control rat IgG2a (each 2 µg/mL) for 30 min and then treated with platelets (~ 2 × 10^7^/mL) plus EGTA (2 mM) for 18 h. The RNA was extracted, and the expression of the EMT-related genes, vimentin (**a**), N-cadherin (**b**), PDPN (**c**), and *PAI*-*1* (**d**), was analyzed by real-time PCR. Values are expressed as the mean ± SEM of 4 culture wells from one representative experiment. The platelet-induced increase was normalized by the untreated cells. ***P < 0.001 by one-way ANOVA and Tukey’s test. **e** Western blot analysis of the effect of NZ-1 on the platelet-induced expression of Vimentin. TE11A cells or original TE11 cells in 12-well plates were treated with platelets without or with rat IgG2a or NZ-1 for 18 h as above. The wells were then supplemented with a twofold volume of fresh medium and cultured for an additional 2 days. **f** Densitometric analysis of vimentin levels in TE11A cells. The vimentin levels were normalized to platelet treatment alone (none). The data shown are one representative experiment conducted in triplicate cultures and expressed as the mean ± SEM. *P < 0.01, ***P < 0.001 by one-way ANOVA and Tukey’s test

To clarify the role of PDPN in the regulation of EMT gene expression by platelets, a PDPN-neutralizing antibody (clone NZ-1) [33, 34] was added to the TE11A cell culture prior to their exposure to platelets. The inclusion of NZ-1, but not isotype matched control IgG, suppressed the expression levels of vimentin, N-cadherin, PDPN, and *PAI*-*1* after platelet treatment (Fig. 3a–d). The inhibitory effects of NZ-1 at this concentration (2 µg/mL) was almost maximum; no further inhibition was observed with higher concentrations of NZ-1 (10 µg/mL, data not shown), suggesting the presence of a PDPN-independent mechanism for gene expression by platelets. These results suggested that alterations of EMT-related gene expression by platelets are at least in part mediated by platelet interaction with cell-surface PDPN in these cells.

To assess whether the inhibitory effect of the PDPN-blocking antibody was also observed in the original TE11 cells, which contained both PDPN-positive and -negative population (Fig. 1), the same experiment was conducted in this heterogenous cell population (Additional file 1: Fig. S3). In TE11 cells, vimentin and N-cadherin mRNA induction by platelets were only 3- and 2.5-fold, respectively, which were in stark contrast to the > 200- and > 10-fold induction in TE11A cells (Fig. 3). This is possibly due to the high basal expression levels of 70- and sevenfold, respectively (Fig. 1). The effect of NZ-1 in TE11 cells was not prominent as that in TE11A cells. However, NZ-1 partially inhibited PDPN and *PAI*-*1* induction in TE11 cells, whose basal expression levels were comparable to those in TE11A cells (Additional file 1: Fig. S3C, D). Thus, PDPN involvement in response to platelets is not unique to TE11A cells.

To further ascertain PDPN involvement in platelet-induced EMT-related gene expression in TE11A cells, vimentin protein expression was measured. Western blotting demonstrated that NZ-1 also partially attenuated platelet-induced vimentin expression in TE11A cells (Fig. 3e, f). In TE11 cells, however, vimentin protein was observed, and no distinct change was observed by platelet treatment with or without NZ-1 treatment.

### Generation and characterization of PDPN-knockout TE11A cells

**Fig. 4:**
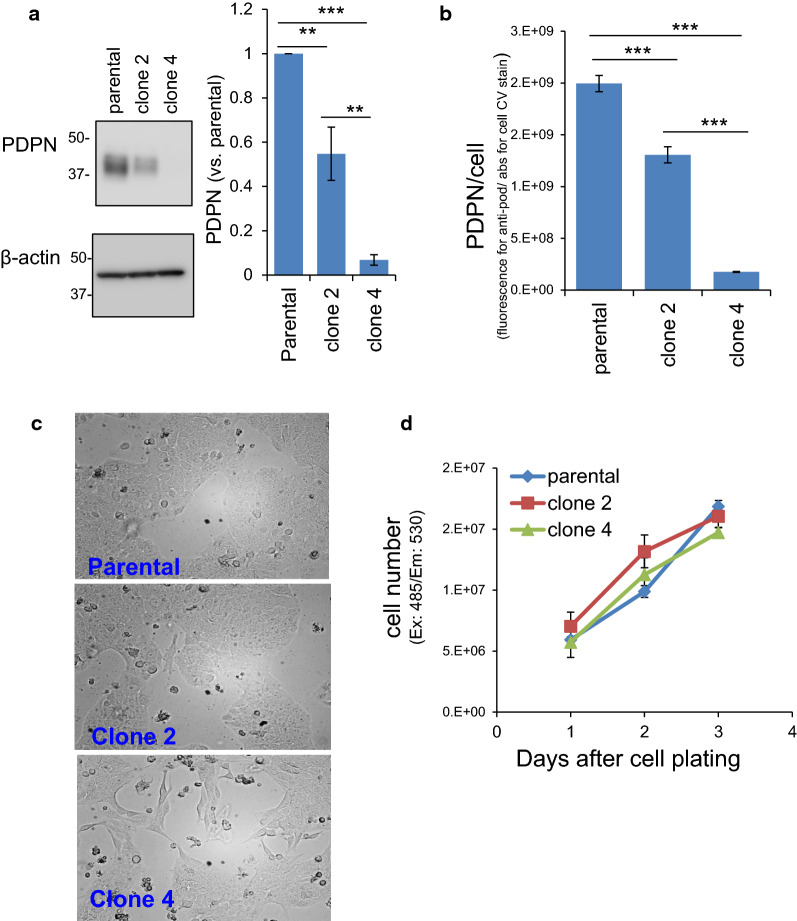
Characterization of PDPN knockout TE11A cells. **a** Western blotting analysis of cellular expression levels of PDPN. The analyses were conducted three times. The densitometric values were normalized to that of parental cells in each assay. Values are expressed as the mean ± SEM. **P < 0.001 and ***P < 0.001 by one-way ANOVA and Tukey’s test. **b** Cell surface expression level of PDPN, assessed by cell-ELISA. Values are expressed as the mean ± intra-assay deviation expressed as the standard deviation (SD) from 4 wells. *** P < 0.001 by one-way ANOVA and Tukey’s test. **c** Proliferation phase morphology of parental, clone 2, and clone 4 TE11A cells grown in culture dishes. **d** Growth rate of clonal cells. Cells were plated in 48-well plates at a density of 2.5 × 10^4^/well. Plates were washed with PBS and frozen on days 1, 2, and 3. After thawing and lysis with 0.5% Triton X-100 in PBS, the nuclei were stained with Cyquant dye and their fluorescence intensity was measured at an excitation and emission wavelength of 485 and 530 nm, respectively. Values are expressed as the mean ± intra-assay deviation expressed as SD for 4 well cultures

To unequivocally clarify the role of PDPN in EMT gene regulation, we knocked out the PDPN gene in TE11A cells using the CRISPR-Cas9-mediated gene ablation method (Fig. 4). Exon 2 of the PDPN gene was disrupted and two clones, clone 2 and clone 4, were successfully isolated. Western blotting analysis revealed that the PDPN protein levels were halved in clone 2 cells and completely undetectable in clone 4 cells (Fig. 4a). RT-PCR analysis of the steady-state PDPN mRNA revealed that clone 2 cells expressed almost half the level of the parental cells and that clone 4 cells had further diminished quantities of PDPN mRNA (Additional file 1: Fig. S4A, also see Fig. 6e) consistent with the protein levels. Immunostaining of cell-surface PDPN molecules also demonstrated a partial (~ 30%) reduction in clone 2 cells and almost complete absence in clone 4 cells (Fig. 4b), consistent with the western blot results of the total PDPN levels. These results demonstrated that clone 2 is partially deficient and clone 4 is completely deficient in steady-state PDPN expression both at mRNA and protein levels, although the underlying silencing mechanism is not straightforward (see discussion).

Morphological observations showed that clone 2 cells were indistinguishable from their parental cells, whereas clone 4 cells showed a fibroblast-like appearance (Fig. 4c). There was no difference in the proliferation rate among the three clonal cells (Fig. 4d).

### Effects of PDPN knockout on platelet aggregation

**Fig. 5:**
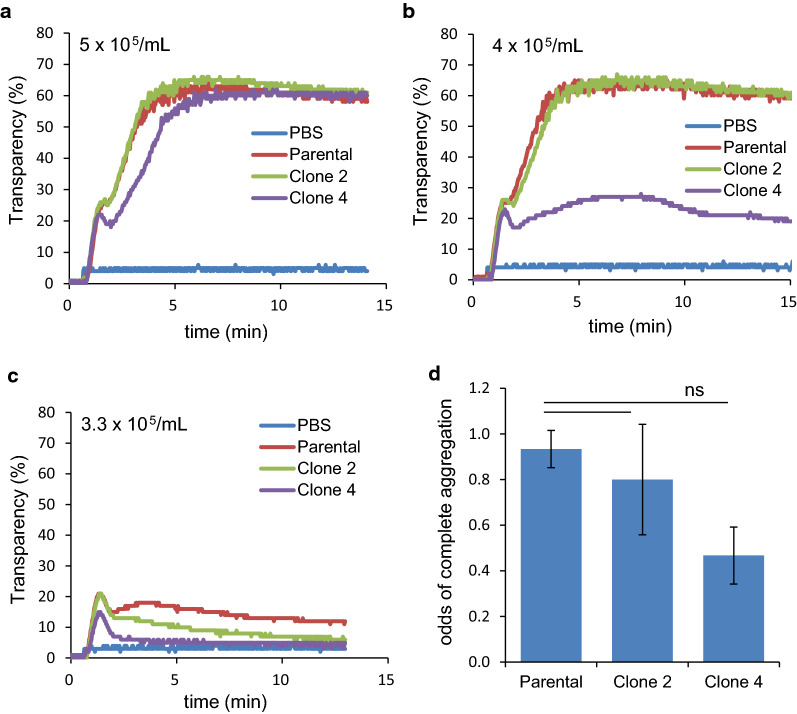
Platelet aggregation-inducing activity of PDPN knockout TE11A cells. In each set of assays (**a**–**c**), the same number of parental TE11A, clone 2, or clone 4 cells, or PBS (vehicle) was added to platelet-rich plasma samples in cuvettes on a 4-channel aggregometer. Aggregation was monitored by the change in turbidity. Traces shown are one representative result obtained at the final cell density of 5 × 10^5^/mL (**a**), 4 × 10^5^/mL (**b**), or 3.3 × 10^5^/mL (**c**). In **d**, the odds for successful full aggregation (transparency > 60% as in **a**), induced by the threshold number of cells (3.3–10 × 10^5^/mL) is summarized from a total of 10 assay sets from three independent experiments. There were no significant differences

To determine whether the decreased levels of cell-surface PDPN affected the phenotypes of theses tumor cells, platelet aggregation assays were performed. Platelet aggregation by parental TE11A cells showed an all-or-none response. The onset of aggregation occurred without a lag time and proceeded to completion if sufficient cells were present. If the cell number was insufficient, incomplete aggregation was observed. Figure 5 shows a typical set of platelet aggregation assay results. When added to PRP samples, all TE11A cell clones, even PDPN-null clone 4, promptly induced robust platelet aggregation, without a lag time (Fig. 5A). However, a slight decrease (5 × 10^5^/ml → 4 × 10^5^/ml) in the number of cells resulted in an incomplete aggregation for clone 4 cells (Fig. 5b). A further decrease (4 × 10^5^/ml → 3.3 × 10^5^/ml) in cell number resulted in an incomplete aggregation for all cells, although the extent of aggregation differed in the same manner as PDPN expression levels, i.e., parental cells > clone 2 cells > clone 4 cells (Fig. 5c). A delay or lag time in the onset of aggregation was not observed after lowering the cell number within our assay time frame of 30 min. Moreover, the anti-PDPN NZ-1 antibody had no inhibitory effect on platelet aggregation by parental TE11A cells (not shown). The odds for the complete aggregation of each TE11A cell clone are summarized in Fig. 5d, with data obtained from three independent experiments. These results demonstrate a tendency, although not statistically significant, that cell-surface PDPN expression levels are correlated with the platelet aggregation activity of cells around their cell density threshold. Above the cell density threshold, however, mechanisms other than PDPN become dominant in platelet aggregation by all the TE11A cell clones.

### Effects of PDPN knockout on EMT gene expression

**Fig. 6:**
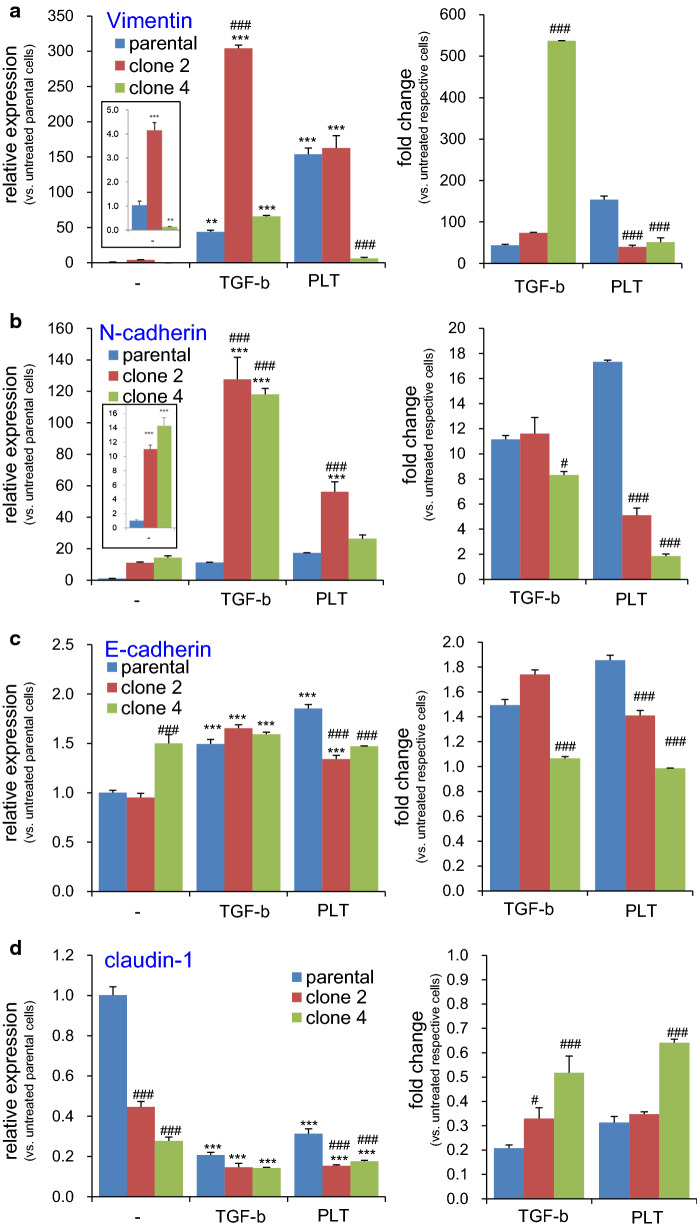

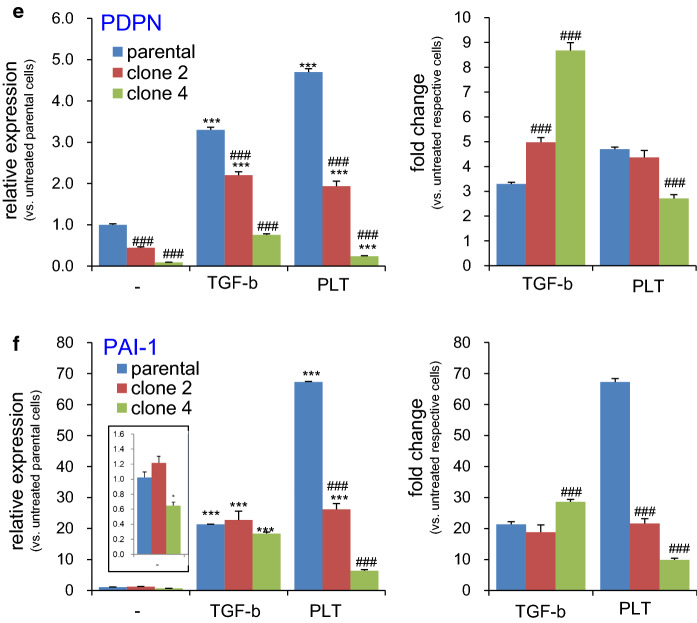
Effect of PDPN knockout on PLT- or TGF-β-induced expression of EMT-related genes in TE11A cells. Parental, clone 2, or clone 4 TE11A cells at confluence in 24-well plates were treated with TGF-β (20 ng/mL) or platelets (~ 2 × 10^7^/mL) plus EGTA (2 mM) for 18 h. The RNA was then extracted, and the expression of the EMT-related genes, vimentin (**a**), N-cadherin (**b**), E-cadherin (**c**), claudin-1 (**d**), PDPN (**e**), and *PAI*-*1* (**f**), was analyzed by real-time PCR. In each left side graph, relative level of expression is shown with respect to non-treated parental TE11A clone, whereas in the right side graph, fold induction is expressed with respect to the basal level of each clonal cells. The inset graph in (**a**, **b**, **f**) shows the magnified bars for the basal level of expression of each cell clone. Values represent the mean ± SEM of quadruplicate culture wells from one representative experiment. *P < 0.001 and ***P < 0.001 vs. non-treated parental cells by one-way ANOVA and Tukey’s test, ^###^P < 0.001 vs. parental cells of respective treatment group by two-way ANOVA and Bonferroni’s test

Next, we measured the impact of PDPN deficiency on EMT-related gene expression. Profound alterations in the basal levels of some EMT-related gene expression was observed, and, therefore, relative expression levels of each gene were calculated with respect to those in the untreated parental cells (Fig. 6, left graphs). PDPN deficiency resulted in N-cadherin upregulation (Fig. 6b inset) and claudin-1 downregulation (Fig. 6d) in both clone 2 and clone 4 cells compared to those in the parental cells. The partially PDPN deficient clone 2 cells, but not clone 4 cells, also showed elevated basal expression levels of vimentin (Fig. 6a inset). These alterations engendered by PDPN deficiency indicate EMT progression, contrary to the findings that elevated PDPN levels favor EMT [6, 12, 14, 15].

These clonal cells were treated with TGF-β or platelets to determine the role of PDPN on the induced expression of EMT-related genes. To focus on stimulus responding-capacity of each clonal cells, fold-induction was also expressed with respect to the basal level in respective clones (Fig. 6, right graphs). Consistent with the effects of NZ-1 (Fig. 3), the fold increase of vimentin, N-cadherin, and *PAI*-*1* over the basal level in each clone cells was severely suppressed in partially PDPN deficient clone 2 cells, and, to a further extent, in PDPN-null clone 4 cells. Nevertheless, TGF-β-induced expression of these genes in either clone cells were largely intact or even potentiated, demonstrating the specific involvement of PDPN in platelet-dependent EMT gene expression. It is worth noting, although in small levels, that TGF-β, and to a further lesser extent, platelets, also increased PDPN expression in completely PDPN deficient clone 4 cells. These results confirm that clone 4 cells were not quintessential homozygous knockout cells, and suggest that the PDPN mRNA expression was somehow suppressed (see discussion section).

**Fig. 7:**
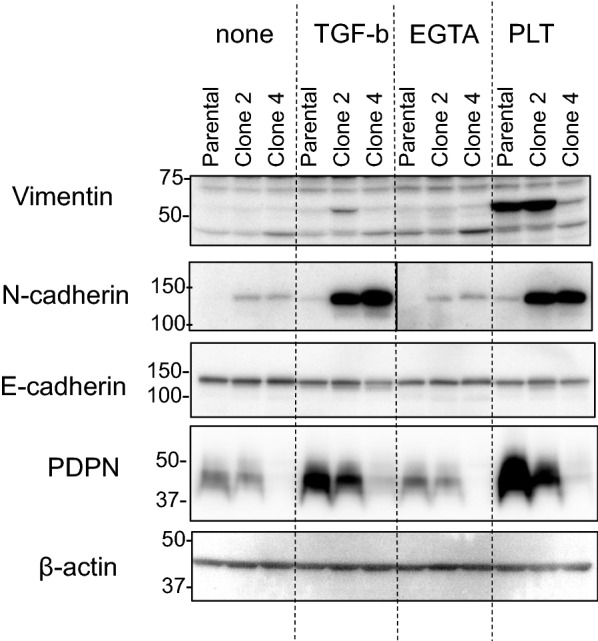
Effect of PDPN knockout on PLT- or TGF-β-induced expression of EMT-related proteins in TE11A cells. Cells in 6-well plates were treated with TGF-β (20 ng/ml) or platelets (~ 2 × 10^7^/ml) for 24 h, followed by supplementation of fresh medium and an additional 2 days of culture. Cellular proteins were analyzed by western blotting using the indicated antibodies. One set of representative results is shown

To confirm the changes in the EMT-related gene expression profile in PDPN-deficient cells at the protein level, the levels of selected proteins were measured by western blotting. As shown in Fig. 7, without any treatment, vimentin was undetectable in either clone cells. Treatment with TGF-β induced vimentin protein expression, preferentially in clone 2 cells in accordance with hyper-elevation of its mRNA in this clone cells. In contrast, treatment with platelets induced large amounts of vimentin protein, both in the parental and clone 2 cells. However, platelets failed to induce vimentin protein expression in PDPN-null clone 4 cells, which was also consistent with mRNA results. Under non-treated basal conditions, N-cadherin was clearly detectable in both clone 2 and clone 4 cells, consistent with spontaneous elevation of its mRNA in both clones. Treatment with TGF-β or platelets robustly augmented N-cadherin levels in both clones. The levels of E-cadherin protein were almost unchanged in any clone cells regardless of EMT-inducing treatments, consistent with the mRNA results, as well as the results reported in a previous study [14]. TGF-β and platelets also increased PDPN protein expression in the parental and clone 2 cells, however, the levels were proportionally reduced in half-deficient clone 2 cells, consistent with the mRNA results. It is worth noting that TGF-β and, to a smaller extent, platelets also induced small amounts of PDPN protein in otherwise PDPN-null clone 4 cells, consistent with the mRNA levels in these cells. Overall, the protein expression levels in these clone cells were similar to the relative mRNA levels in these cells.

### Effect of re-introduction of PDPN gene on N-cadherin levels

One of the characteristics of PDPN knockdown is the spontaneous expression of N-cadherin protein. As such, next, we investigated whether N-cadherin upregulation was a reversible phenotype. To this end, a cytomegalovirus promoter-driven PDPN-expression vector or its empty vector was introduced into each TE11A subclone cell using lentivirus, and PDPN was stably expressed. Nearly all the cell populations were transfected with the expression vector as judged by venous-positive under fluorescence microscopy (data not shown). The levels of N-cadherin and PDPN were measured at the protein level on day 7 and day 12 after transfection (Additional file 1: Fig. S5). Although significant amounts of PDPN were stably expressed in each clone cell, the N-cadherin levels were not affected. The ectopic expression of PDPN has been demonstrated to cause a reduction in E-cadherin in some cells [12, 13]. However, the E-cadherin levels were also unaltered in all clone cells. These results demonstrated that N-cadherin induction by PDPN deficiency is an irreversible response as observed in cellular differentiation stages in general.

### Effects of PDPN knockout on invasion ability and motility

**Fig. 8:**
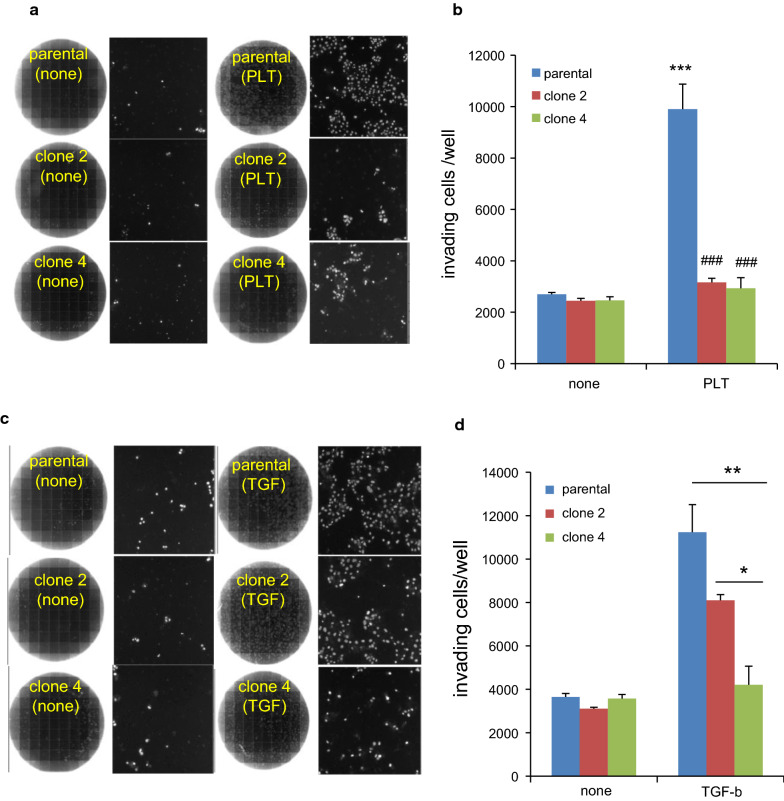
Effect of PDPN knockout on invasion ability. **a**, **c** Composite image of an entire trans-well (left) from a total of 196 images and one of representative images showing DAPI-stained cells (right). Cells were treated with platelets plus EGTA (A) or TGF-β (C) for 1 day, followed by supplementation with fresh medium and additional culturing for 2 days. On day 3, the cells were harvested and seeded in the upper matrigel chambers with serum-free medium (1.5 × 10^5^). The chambers were then placed in holder wells containing 10% serum-containing medium. Two days after the trans-well culture, the number of invading cells was counted using ArrayScan, as described in the Materials and Methods. **b**, **d** Histogram of the number of invading cells for each clone cells with or without EMT induction. In **b**, the assay was conducted in quadruplicates and the values are expressed as the mean + SEM. **P < 0.001 and ***P < 0.001 vs. non-treated parental cells by one-way ANOVA and Tukey’s test, and ^###^P < 0.001 vs. parental cells of respective treatment group by two-way ANOVA and Bonferroni’s test. In **d**, the invasion of non-treated cells was measured in duplicate and the values are expressed mean ± range, while those for TGF-β-treated group triplicates is expressed as the mean ± SEM by one-way ANOVA and Tukey’s test

The effects of PDPN deficiency on the tumor invasive ability were assessed using 8-µm membrane trans-well chambers. Cells were treated with or without platelets for 3 days before transferring to the chambers (Fig. 8). Without EMT induction, there was no difference in the number of invading cells among the clone cells, indicating that at the resting state PDPN level itself had no effect on the cellular invasion ability. In contrast, after treatment with platelets, the number of invading cells in the parental cells increased by almost fourfold. However, platelet-induced invasion was almost completely absent in both clone 2 and clone 4 cells. Similarly, invasion after TGF-β treatment was also partially suppressed in clone 2 and almost completely suppressed in clone 4 cells. These results clearly demonstrate that the PDPN expression levels affect the invasion ability of TE11A cells after EMT regardless of its inducer. The profound potentiating effect of PDPN levels on cellular invasion capacity after EMT induction was also consistent with the results of siRNA-mediated PDPN silencing in original TE11 cell population after TGF-β treatment [14].

**Fig. 9:**
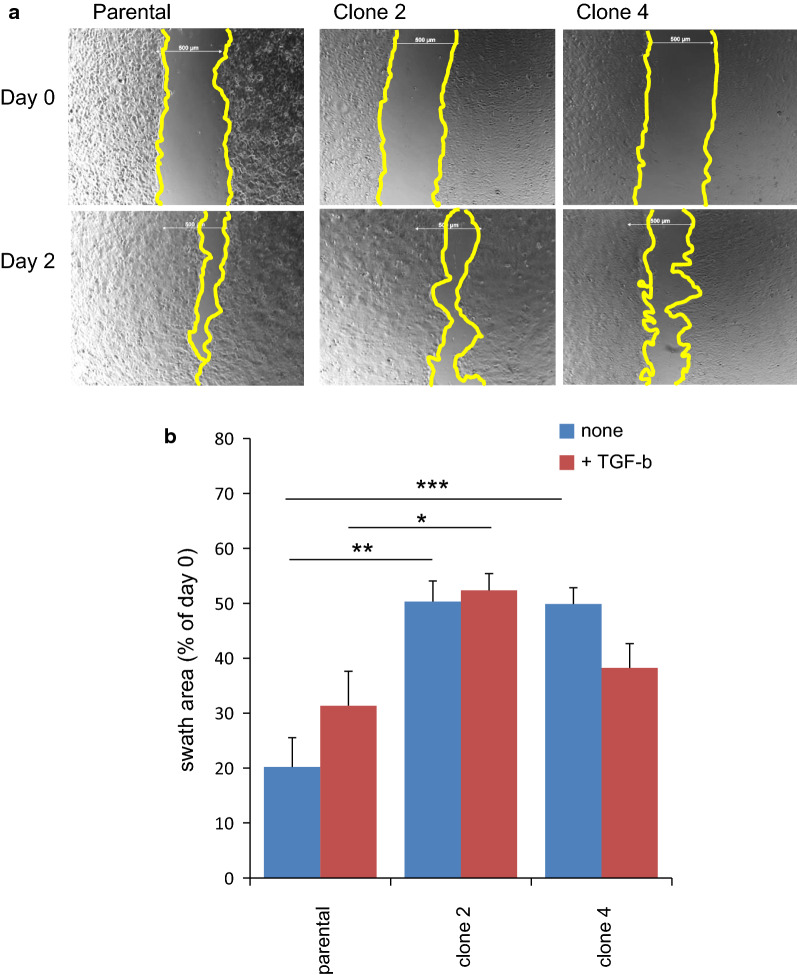
Effect of PDPN knockout on wound healing. **a** Representative image of TE11A cells in 24-well plates after denudation and the corresponding scratched positions at 48 h after recovery culture without TGF-β. Scale bar = 500 µm. The edges of the swath areas have been circumscribed for clarity. **b** Summary of the swath area of respective TE11A cells after 48 h of recovery culture, with and without TGF. Values are expressed as the mean ± SEM of a total of 6 positions from 3 wells. *P < 0.05, **P < 0.01 by one-way ANOVA and Tukey’s test. One set of representative results is shown

Next, we measured cellular motility using a wound healing assay (Fig. 9). The rate of cell migration into the denuded area, for both clone 2 and clone 4 cells, was half that of the parental cells, regardless of the presence of TGF-β. These results demonstrated that PDPN plays an important role in the migration of TE11A cells.

## Discussion

The major findings of this study were as follows: (i) PDPN-knockout TE11A cells, both the half-deficient clone 2 and completely deficient clone 4, were still able to induce platelet aggregation; (ii) PDPN knockout in either allele resulted in the spontaneous upregulation of N-cadherin, both at the mRNA and protein levels, and complete deficiency changed the morphology of the cells to a fibroblast-like appearance; (iii) Even half-deficiency of cell-surface PDPN attenuated platelet-induced EMT-related gene expression, including vimentin, N-cadherin and PAI-1 without affecting TGF-β-induced expression of these genes; (iv) a PDPN-neutralizing antibody also suppressed the platelet-induced upregulation of EMT-related gene expression; (v) PDPN deficiency itself did not affect the basal invasion capacity but impaired its elevation after EMT induction; (vi) PDPN deficiency, whether half or complete, negatively affected cell motility.

### Role of PDPN in cellular phenotype and platelet activation

PDPN has been previously found to bind to CLEC-2 on the platelet surface to induce platelet aggregation [23, 35]. However, in this study, we found that TE11A cells completely deficient in PDPN were still able to induce platelet aggregation, with similar aggregation kinetics to the parental cells. We concluded therefore that, in TE11A cells, the role of PDPN in platelet aggregation is marginal. Besides the PDPN-CLEC-2 axis, several interactions with cell surface molecules have been reported for tumor cell-induced platelet aggregation, including interactions between ADMA10, galectin-3,α_V_ β_III_ and CD97 on tumor cells and α_6_β_1_, gpVI, α_IIb_β_III_ and an unidentified target on platelets, respectively [18, 20, 36, 37]. In addition to direct contact, tumor-secreted factors such as thrombin, matrix metalloproteinases and ADP have also been suggested to cause platelet aggregation [18, 36, 38]. Studies are currently underway to elucidate the mechanisms of platelet aggregation by TE11A cells.

The experimental manipulation of PDPN expression levels has shown that PDPN is positively coupled with mesenchymal marker gene expression in many cells [6, 12, 14, 15]. In this study, however, we observed a shift in the seemingly opposite direction regarding EMT marker gene expression by PDPN knockout, among others, with the spontaneous expression of the mesenchymal marker, N-cadherin. To our knowledge, N-cadherin upregulation by decreased PDPN levels is a very rare phenotypic alteration, although not unique; the experimental results of Takeuchi et al. [13] also showed that shRNA-mediated downregulation of PDPN in pleural mesothelioma H226 cells also caused the spontaneous expression of N-cadherin, along with vimentin proteins, although the authors did not mention these changes. Regarding the morphology, a previous study showed that PDPN overexpression caused a shift into mesenchymal-like morphology in MDCK cells [12]. In our TE11A cells, conversely, a complete deficiency in PDPN expression caused clone 4 cells to take a mesenchymal-like morphology, consistent with N-cadherin expression. PDPN seemed to play a role in maintaining an epithelial-cell like morphology in this cell line, consistent with the observation that scattered cells with mesenchymal-like morphology in the original TE11 cell population were PDPN-negative. This drastic change in cell morphology caused by PDPN deficiency in TE11A cells could be attributed to the effect of actin filament re-arrangement, which PDPN regulates via binding with REM proteins (ezrin, radixin, and moesin) [12, 16]. Perhaps the impact of PDPN levels on EMT marker gene expression and actin cytoskeletons is not straightforward, but has cell-type and context dependencies. Further studies will be needed to clarify these issues.

Apart from EMT markers, PDPN knockout, even half-deficiency, resulted in a decreased motility of TE11A cells in wound-healing assays, consistent with a number of studies showing a positive correlation between PDPN levels and cell motility using the same assay [6, 12–14, 39]. Unlike these cells [6, 12–14, 16, 39], however, in matrigel invasion assay without EMT induction, the PDPN levels had no effect on the invasion ability of the resting state of TE11A cells. The effect of PDPN levels on the invasion ability of this cell line became apparent after the cells were induced by platelets or TGF-β for EMT. In this regard, Sikorska et al. [40] recently demonstrated that PDPN knockdown in a thyroid cancer line potentiated basal invasion ability and suggested that whether PDPN levels affect matrigel invasion is dependent on the types of cells. Our present results further indicate that the invasion capacity of tumor cells is synergistically controlled by PDPN levels and EMT inducers.

### Role of PDPN in EMT gene expression by platelets

We propose that the major role of PDPN in platelet-induced EMT gene expression is to elicit TGF-β secretion from platelets. The similarity in the mRNA expression profiles of EMT-related genes in parental TE11A cells by platelets and by TGF-β treatment, and the complete inhibitory effect of SB431542 in both cases, suggests that the platelet effects are largely mediated by TGF-β derived from activated platelets, as suggested from by studies using mouse platelets [15, 17]. Platelet-induced EMT-related gene expression was also inhibited by anti-PDPN antibody (NZ-1) and by complete PDPN deficiency (clone 4), suggesting that the release of TGF-β from platelets could be significantly regulated by the PDPN-CLEC-2 interaction, as suggested [15]. Since the contribution of PDPN in platelet aggregation by TE11A cells is marginal, TGF-β release from platelets by PDPN interaction is considered to be a rather specific reaction. Thus, TGF-β is an entity of PDPN-dependent, platelet-induced EMT-related gene expression in TE11A cells, at least at the mRNA level.

However, there is a decisive difference between the actions of TGF-β and of platelets in EMT-related gene expression in TE11A cells. While platelets could potently induce vimentin protein expression, TGF-β alone in TE11A parental cells could not, despite both treatments resulting in almost comparable levels of vimentin mRNA expression. This disparity suggests that vimentin mRNA translation requires an additional signaling input to TGF-β signaling in this cell line. A previous study suggested that platelets activate two signaling pathways in some tumor cells, leading to vimentin protein expression; one is the platelet-tumor contact-dependent activation of NF-κB signaling and the other TGF-β signaling [17]. A similar two-hit mechanism could be applicable to TE11A cells. One possible such additional signaling pathway activated is a signal from the PDPN cytosolic domain, which involves the ezrin-dependent activation of Rho A kinases and actin filament re-arrangement [3, 12]. An alternative possibility is the presence of additional bioactive factors released from platelets in a PDPN-CLEC-2 interaction-dependent manner. Sphingosine 1-phosphate (S1P) is one such PDPN-inducible bioactive molecule from platelets [4] and has an EMT inducing activity [41].

**Fig. 10:**
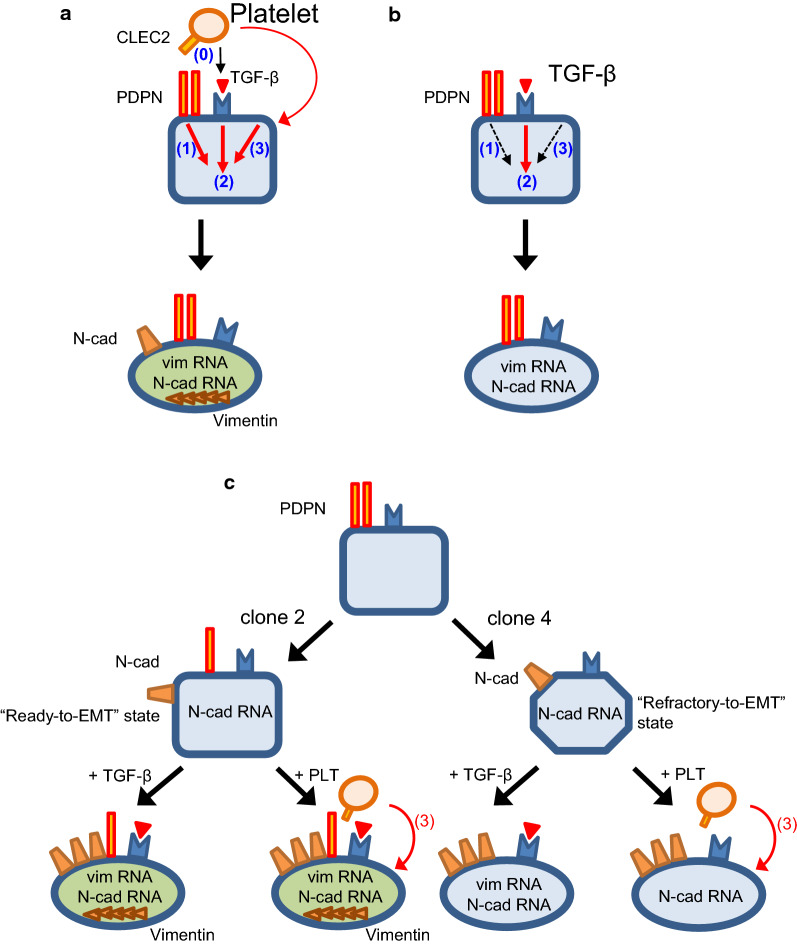
Schematic model for the role of PDPN in EMT-related gene expression in TE11A cells. **a** In TE11A cells, at least three signaling pathways (signaling module 1–3) are involved in the induction of EMT gene expression. The binding of PDPN to CLEC-2 activates signaling module 1 and results in the release of TGF-β from platelets (event 0), which in turn activates signaling module 2. Platelets also activate other signaling pathways (signaling module 3). The activation of signaling module 2 plus module 1 and/or 3 synergistically induces the expression of Vimentin and N-cadherin proteins. **b** Potential signaling pathways activated by TGF-β. TGF-β activates only signaling module 2, resulting only in the mRNA expression of Vimentin and N-cadherin. **c** The impact of PDPN knockout on EMT gene expression at resting state and after treatment with TGF-β or platelets. A decrease in the PDPN levels causes spontaneous N-cadherin protein expression, decrease in claudin-1 expression, and prepares the cell to transition into a mesenchymal state (“Ready-to-EMT”) fully in clone 2 cells, but incompletely in clone 4 cells. Half-deficient PDPN clone 2 cells can still interact with platelets and further undergo EMT, however, completely PDPN deficient clone 4 cells cannot (see “Discussion” for more details)

Based on the present results and data interpretation, the following schematic could be envisioned for the role of PDPN in EMT-related gene expression in TE11A cells (Fig. 10a). The interaction of platelets and TE11A cells is possible in multiple ways. Among them, PDPN-CLEC-2 interaction results in a significant release of TGF-β from platelets (event 0). There are at least three signaling pathways (signaling module 1–3) in TE11A cells that are associated with the induction of EMT gene expression. The signaling module 1 is constituted of PDPN and its associated proteins and, upon binding with CLEC-2, transmits a signal from its cytosolic domain. The signaling module 2 is TGF-β receptor signaling pathway and is activated by TGF-β from platelets. All the other signaling pathways activated by platelets are collectively referred to as signaling module 3, which possibly includes platelet-cancer cell direct contact [17, 20], platelet-derived lysophosphatidic acid [37], S1P [4], PDGF, and microRNA939 [36]. Upon interaction with PDPN, platelets activate signaling module 1 and/or module 3 in parallel with signaling module 2, thereby resulting in the expression of N-cadherin and Vimentin proteins. In contrast, the activation of signaling module 2 alone by TGF-β addition resulted in the mRNA expression of vimentin and N-cadherin, however, not in their protein expression (Fig. 10b).

Decreased PDPN protein levels resulted in spontaneous N-cadherin protein expression and primed the half-PDPN deleted clone 2 cells toward transition into a mesenchymal-like state (“Ready-to-EMT” state) (Fig. 10c). Therefore, in clone 2 cells, the activation of the signaling module 2 alone by TGF-β can induce vimentin protein expression without input into signaling module 1, as well as the super-induction of N-cadherin. Complete PDPN-deficient clone 4 cells were also predisposed to the “Ready-to-EMT” state, however, to a lesser extent than in clone 2 by unknown mechanism. N-cadherin protein is also upregulated in clone 4 cells and is further augmented by TGF-β. Because of the incomplete priming nature, however, vimentin mRNA level after TGF-β treatment is the same as in the parental cells, and never accomplishes protein expression as observed in clone 2 cells. Clone 4 cells can still induce platelet aggregation in a PDPN-independent manner, and, therefore, activated platelets could in turn trigger signaling module 3, thereby resulting in the further accumulation of N-cadherin protein. However, the lack of PDPN-CLEC-2 interaction cannot mobilize TGF-β from platelets (event 0), such that the signal input into both module 1 and module 2 is diminished. For these mechanisms, complete PDPN-deficiency, as well as treatment with anti-PDPN neutralizing antibody NZ-1, results in attenuated vimentin expression both at the mRNA and protein levels. Finally, because of the negative effects of decreased PDPN levels on actin filament rearrangement, which overwhelm the advantages of the “Ready-to-EMT” status for cellular motility, PDPN deficiency overall abrogates platelet- or TGF-β-induced potentiation of invasion ability.

### Potential mechanism of PDPN downregulation

In this study we generated two different PDPN-deficient clonal cells, clone 2 and clone 4, from a subclone of TE11 cells (TE11A cells), expressing almost half and undetectable, respectively, levels of PDPN at mRNA and protein levels compared to those in the parental TE11A cells. Clone 2 cells had a 250-bp deletion in one allele (Additional file 1: Fig. S4C) and an intact allele, and, therefore, were technically a heterozygous gene knockout. Clone 4 cells also had a 250-bp deletion in one allele. In the other allele, however, we were unable to identify any indels around the Crispr target site or even in the extension of a 1600 bp region (Additional file 1: Fig. S4C). In a quintessential Crisper-mediated knockdown, mRNA level from a mutated gene is expected to be the same as the intact gene. Therefore, the basal PDPN mRNA in clone 4 is expected to be the same as that in clone 2 cells. However, it was severely reduced (Additional file 1: Fig. S4A).

Since clone 4 cells can still induce PDPN mRNA upon TGF-β stimulation, the decreased PDPN mRNA level in these cells could be due to increased mRNA instability. There would be yet-to-be identified mutation(s) distant from the target site in the “intact” PDPN allele as a consequence of the “on-target” effect. A recent study has shown that CRISPR-Cas9-mediated gene knockout may cause large deletions and genomic rearrangements in the distal region, even several kilobase apart, as a consequence of the “on-target” Cas9 cleavage effect [42]. In fact, both clone 2 and 4 cells have a 250 bp deletion, indicating that the cleavage had occurred 210 bp upstream and 40 bp downstream from the Crispr target site. The identification of the molecular mechanism of PDPN downregulation in clone 4 would contribute to the as-of-yet unsolved “on-target” silencing mechanisms of Crispr-Cas 9 gene editing technology [42, 43].

## Conclusion

The present study demonstrates that PDPN is essential for the platelet-induced expression of certain EMT-related genes in TE11A cells. The absence of PDPN resulted in the spontaneous upregulation of N-cadherin and shifted the propensity of the cells in favor of EMT-related gene expression. Nevertheless, a lack of PDPN significantly impairs cellular motility and EMT-associated invasiveness in TE11 cells.

## Supplementary information

**Additional file 1: Fig. S1.** Effect of EGTA on PLT-induced vimentin expression in TE11A cells. TE11A cells at confluence in 24-well plates were treated with platelets (~ 2 × 10^7^/mL) or TGF-β (20 ng/mL), in the presence of the indicated final concentrations of EGTA for 18 h, and vimentin expression was measured by real-time PCR. EGTA was added to the culture just before the addition of platelets or TGF-β. Values are mean ± range of duplicate culture wells from one representative experiment. **Fig. S2.** Effect of SB431542 on PLT- or TGF-β-induced expression of EMT-related genes in TE11A cells. TE11A cells at confluence in 24-well plates were preincubated with 0.1% DMSO (control) or 5 µM SB431542 for 30 min and then treated with TGF-β (20 ng/mL) or platelets (x1 = ~ 0.7 × 10^7^/mL, ×3 = ~ 2 × 10^7^/mL) plus EGTA (2 mM) for 18 h. The expression of the EMT-related genes, vimentin (A), N-cadherin (B), E-cadherin (C), claudin-1 (D), PDPN (E), and *PAI*-*1* (F) was analyzed by real-time PCR. Values are mean ± range of duplicate culture wells from one representative experiment. **Fig. S3.** Effect of the PDPN-neutralizing antibody, NZ-1, on PLT-induced expression of EMT-related genes in original TE11 cells. Original TE11 cells at confluence in 24-well plates were preincubated with NZ-1 or control rat IgG2a (each 2 µg/mL) for 30 min and then treated with platelets (~ 2 × 10^7^/mL) plus EGTA (2 mM) for 18 h. The expression levels of the EMT-related genes, vimentin (A), N-cadherin (B), PDPN (C), and *PAI*-*1* (D) was analyzed by real-time PCR. Values are expressed as the mean ± SEM of 4 culture wells from one representative experiment. The platelet-induced increase was normalized by the untreated cells. ***P < 0.001 by one-way ANOVA and Tukey’s test. **Fig. S4.** Knockout of PDPN gene in TE11A cells by CRISPR-Cas 9 method. (A) RT-PCR analysis of the open-reading frame of mRNA for PDPN and GAPDH. Clone 2 cells expressed almost half the level of the parental cells and that clone 4 cells had barely detectable quantities of PDPN mRNA. There were no mutations in the ORF of the PDPN mRNA from clone 4 cells. (B) PCR amplification of a 780-bp region containing the CRISPR-Cas 9 target site of the PDPN gene from parental TE11A cells, clone 2 cells, and clone 4 cells. DNA sequencing of the lower 500 bp band confirmed a 250 bp deletion, covering the target site in the PDPN gene (see Fig. S4C). However, there were no indels in the upper 780 bp DNA band from either clone 2 or clone 4 cells. (C) Genomic DNA sequence of human PDPN (GenBank NCBI Reference: NC_000001.11). The sequence highlighted in blue with underline indicates the exon 2 region. The sequence marked in green indicates Crspr-Cas-9 target sequence [19]. The sequence marked in gray indicates ~ 250 bp deletion region identified in one allele of clone 2 cells and clone 4 cells. Red arrows indicate PCR primers used for amplification and DNA sequencing. We have checked the 1800-bp region of the intact PDPN gene from clone 4 cells using combinations of these primers but found no indel within this region.

## Data Availability

All relevant data are within the paper and its Supplementary information.

## Abbreviations

CLEC-2: C-type-lectin-like-2
EMT: Epithelial-to-mesenchymal transition
EGTA: Ethyleneglycol bis(2-aminoethyl ether)-N,N,N,N tetraacetic acid
4MUG: 4-Methylumbelliferyl β-D-galactopyranoside
PAI-1: Plasminogen activator inhibitor-1
PDPN: Podoplanin
PLAG: Platelet aggregation-stimulating domains
PRP: Platelet-rich plasma
TGF-β: Transforming growth factor-β
