# Supplementary material for: Podoplanin is indispensable for cell motility and platelet-induced epithelial-to-mesenchymal transition-related gene expression in esophagus squamous carcinoma TE11A cells
Source: Cancer Cell Int. 2020 Jun 23;20:263. doi: 10.1186/s12935-020-01328-2 (PMC7310449; doi:10.1186/s12935-020-01328-2)
Supplement: Supplementary file 1 — Additional file 1: Fig. S1. Effect of EGTA on PLT-induced vimentin expression in TE11A cells. TE11A cells at confluence in 24-well plates were treated with platelets (~ 2 × 10^7^/mL) or TGF-β (20 ng/mL), in the presence of the indicated final concentrations of EGTA for 18 h, and vimentin expression was measured by real-time PCR. EGTA was added to the culture just before the addition of platelets or TGF-β. Values are mean ± range of duplicate culture wells from one representative experiment. Fig. S2. Effect of SB431542 on PLT- or TGF-β-induced expression of EMT-related genes in TE11A cells. TE11A cells at confluence in 24-well plates were preincubated with 0.1% DMSO (control) or 5 µM SB431542 for 30 min and then treated with TGF-β (20 ng/mL) or platelets (x1 = ~ 0.7 × 10^7^/mL, ×3 = ~ 2 × 10^7^/mL) plus EGTA (2 mM) for 18 h. The expression of the EMT-related genes, vimentin (A), N-cadherin (B), E-cadherin (C), claudin-1 (D), PDPN (E), and PAI-1 (F) was analyzed by real-time PCR. Values are mean ± range of duplicate culture wells from one representative experiment. Fig. S3. Effect of the PDPN-neutralizing antibody, NZ-1, on PLT-induced expression of EMT-related genes in original TE11 cells. Original TE11 cells at confluence in 24-well plates were preincubated with NZ-1 or control rat IgG2a (each 2 µg/mL) for 30 min and then treated with platelets (~ 2 × 10^7^/mL) plus EGTA (2 mM) for 18 h. The expression levels of the EMT-related genes, vimentin (A), N-cadherin (B), PDPN (C), and PAI-1 (D) was analyzed by real-time PCR. Values are expressed as the mean ± SEM of 4 culture wells from one representative experiment. The platelet-induced increase was normalized by the untreated cells. ***P < 0.001 by one-way ANOVA and Tukey’s test. Fig. S4. Knockout of PDPN gene in TE11A cells by CRISPR-Cas 9 method. (A) RT-PCR analysis of the open-reading frame of mRNA for PDPN and GAPDH. Clone 2 cells expressed almost half the level of the parental cells and that clone 4 cells had barely detecta [file 12935_2020_1328_MOESM1_ESM.pdf]

Effect of EGTA on PLT- or TGF-β-induced expression of vimentin in TE11A cells

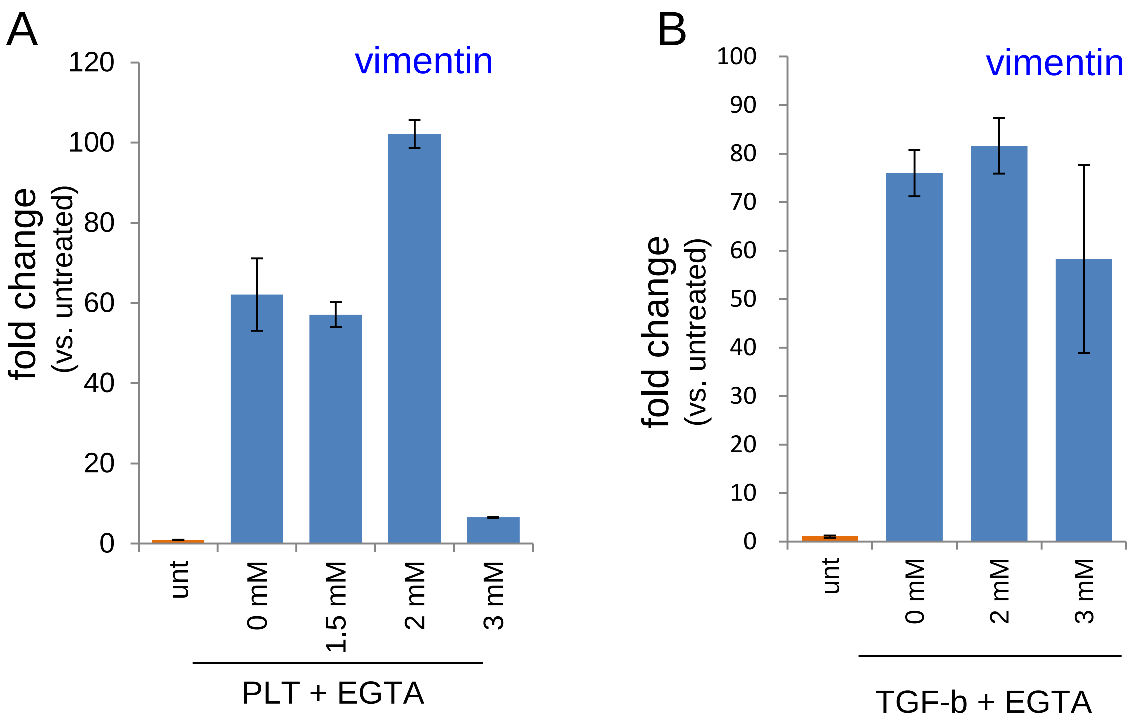

Supplemental Fig. 1

Effect of SB431542 on PLT-or TGF-β-induced expression of EMT genes in TE11A cells

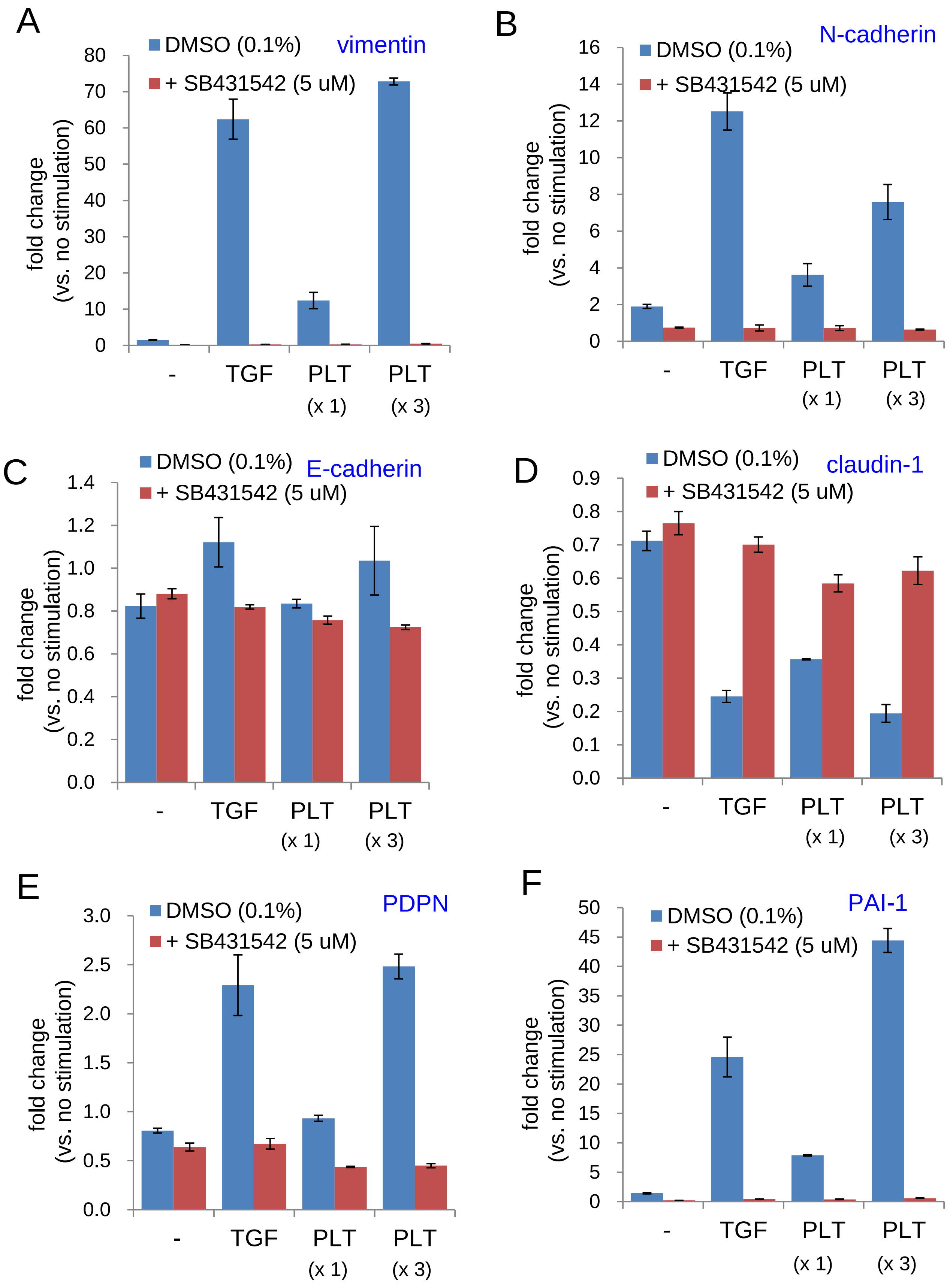

Supplemental Fig. 2

Effect of NZ-1 on PLT-induced expression of EMT-related genes in original TE11 cell populations

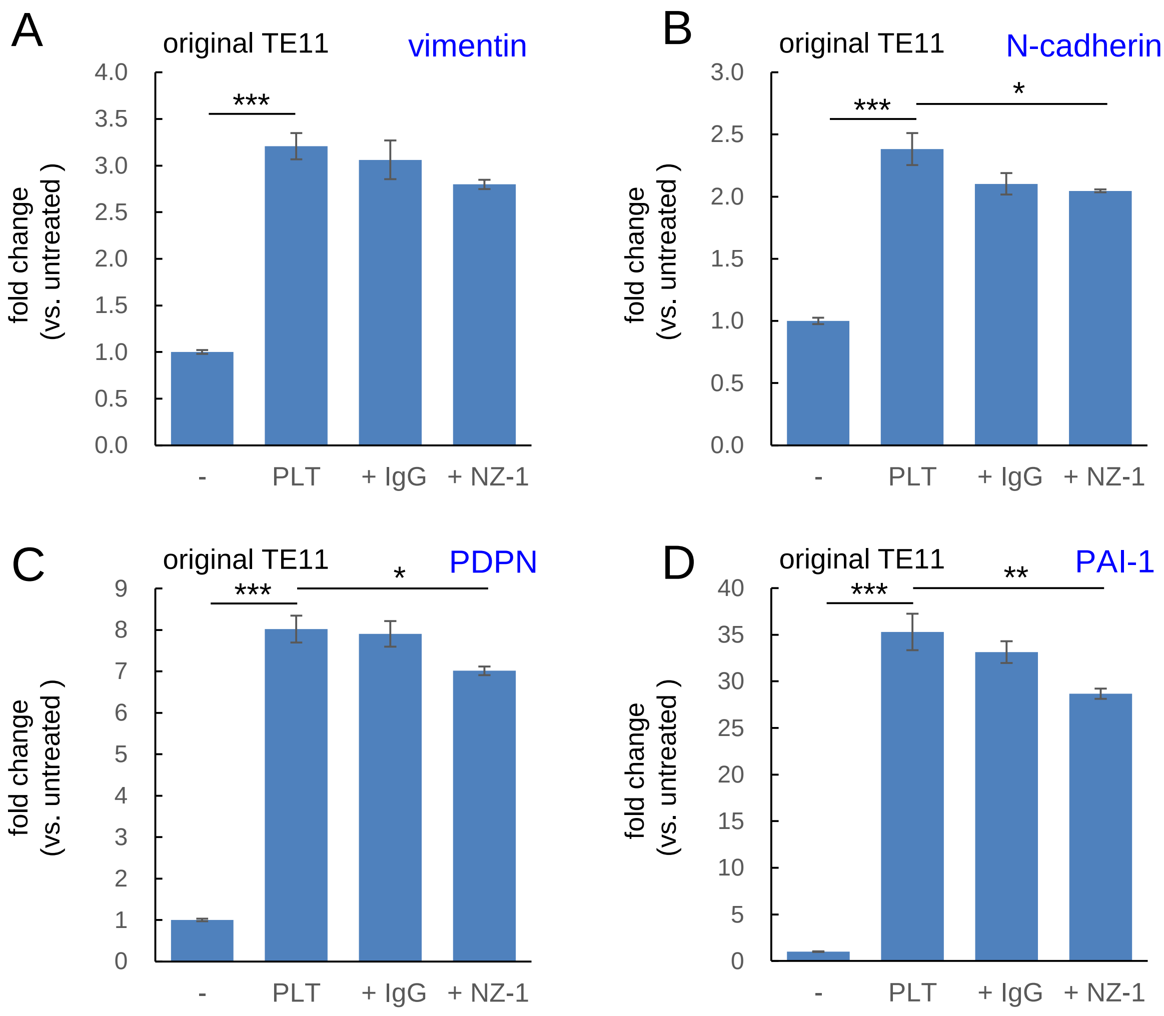

Supplemental Fig. 3

Generation of PDPN knockout TE11A cells

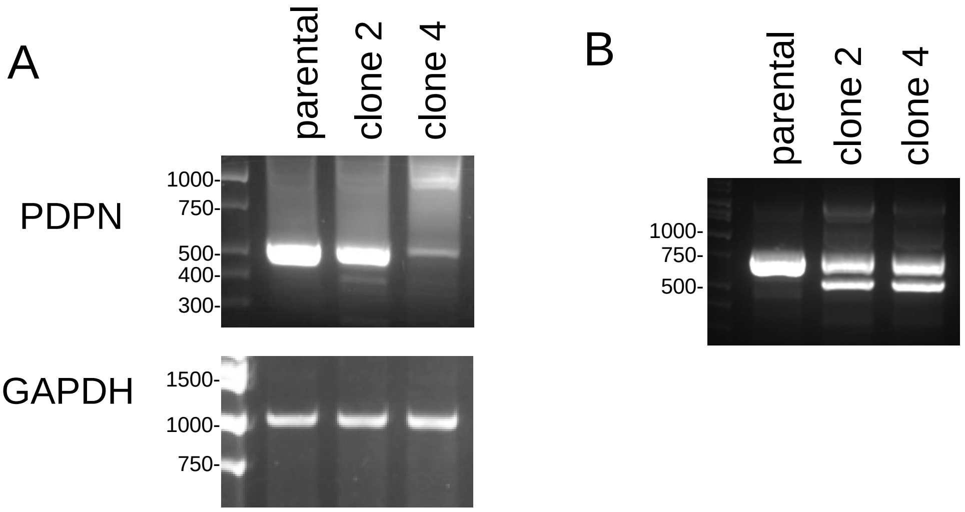

C

```
22621 tagacagtaa tctcaactgt aaagtaagag gtaaaactcc atgtaatcta cgaggtctgt   Forward 3
22681 ggtcagagcc tgtggttcta attgtccaag tgaacagcag tcttaaaatt aaatggagag
22741 agagtttgct tttaaaaaga tggaagttgg gtgtggtggt gagcacttgt agtccccgct
22801 actttggaga ctgagacggg aggatcgctt gagcccagga gtttgaggtt atagtgagct
22861 atgattgtgc ctctgtactc tagcctcagc aacagaggga gagcctgtct cttaagaaaa
22921 attaatttga aaaggtagag aaaaaaataa aaaaataaaa agatggagac cccgcttttg
22981 catcgggtct gcaaaggggt gtgtttagaa atctttcctg ttgactctaa tttagagttt
23041 ttaggtccag atgaatatat taattcatca tatgaaatga caccaggaag aatctggttc
23101 cttattgaaa aggaaagatg tacgtcccct gtccaggcca cctcgatggg gccccaggga
      Forward 1
23161 ggggctggct gctctacctt ttaatgtttt cattttcctt tgctttgcaa cggagataac   Forward 2
23221 cagggtgttg atgtgtcaga agtgctgcaa ggagaatggt ttggtttctg gcaccacagt   Reverse 3
23281 aagccacaaa aaggaccaaa aataaaacaa aaatttccaa aagaaatgga aaagaaaata
23341 atccgaatgt agccgacaag ttgggtctgc ttatgcaatt tccaccttaa gctctgactc
23401 aatctttctt cttcagccag cacaggccag ccagaagatg acactgagac tacaggtttg
23461 gaaggcggcg ttgccatgcc aggtgccgaa gatgatgtgg tgactccagg aaccagcgaa
23521 gaccgctata agtctggctt gacaactctg gtcagtgtcc tgggaagaga ggaatttttt
23581 ccgtaggcat gtgatcacat gcaaggctat gatgtatatt aatttacttt atataaaaat
23641 atatctatct ataaaatcag catgagccac cataccaagg aatagttacg tggctcaaca
                                                       Reverse 2
23701 aagaagaata agtcctcact taatgttgtt gataggttct tggaaactgt gacttgaagc
23761 aaaatgactt agaaagaacc aacattatgt gaggacctgc tgcatgtcat tttccttaaa
23821 gtcgcagttt ccaagaatct atcaacaacg ttaagtgagg agttactgtc tttcccaatt
Forward 4
23881 tcgtccactt agctggtgtg ctgatcacag gtggttggag agtagaagcc agagcttctc
                          Reverse 1
23941 aaattggtaa aaggagccag gattttacat ttccaatctg ttacagccca actttcaata
24001 caatgaaagt tttcttgggt gttgtggcaa tgtcattgct ataaagggtt ctaaatatat
24061 cttctcagcc gggcgtggtg gctcacacct gtaatcccag cactttggga ggctgaggca
24121 ggtggatcac ctgaggtcag gagttcgaga tcagcctgac caagatgatg aaaccttgtc
24181 tctattaaaa atacaaaaat tagctgggcg tggctgtgtg cacctgtgat cccagctgct
24241 cgggaggctg agacaggaga atagcttgaa cccgggaggt ggaggttgca gtgagccgag
24301 atcgcaccac tgcactccag cctgggcaac gagagtgaaa ctccgcctca aaaaataaat
24361 aaataaataa gtcagtcagt ctcaatttgt tttctcacta tagactggta ataaacagga
24421 ctagaatcag tccacagacc acaagatgat agactattta gcctctcgac caacacattc
Reverse 4
24481 ttaacctgat gaattttaat taactcctca tatgtgttac atacctacca tagacaagag
```

Supplemental Fig. 4

Effect of re-introduction of PDPN gene on the levels of E- and N-cadherin

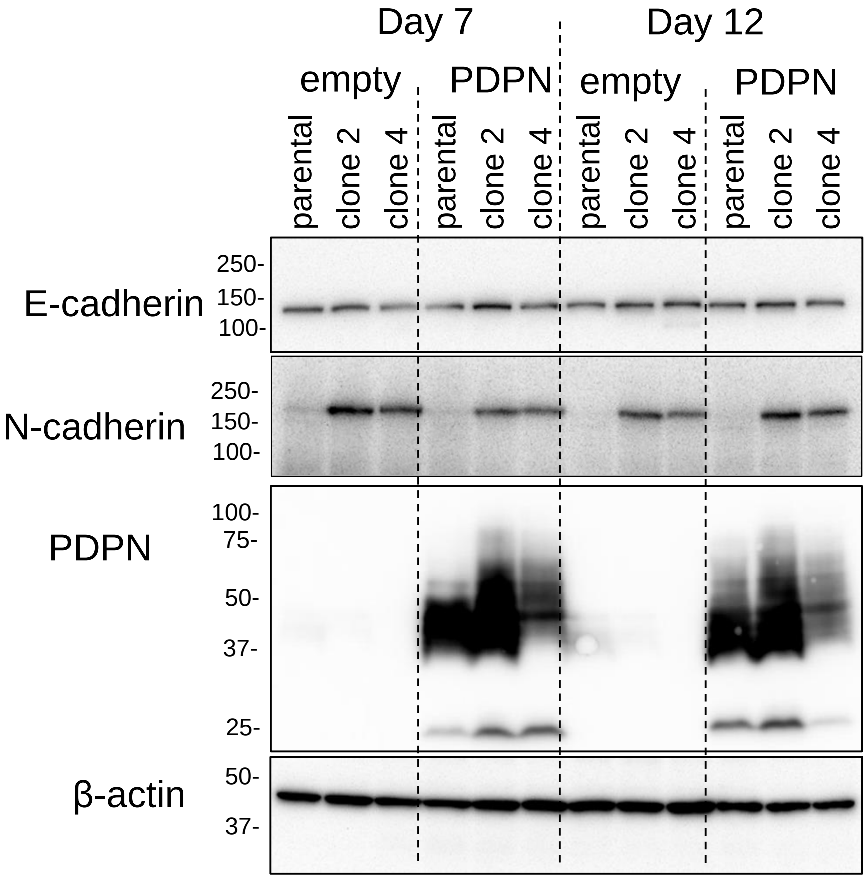

Supplemental Fig. 5
